# Diverse non-canonical electron bifurcating [FeFe]-hydrogenases of separate evolutionary origins in *Hydrogenedentota*

**DOI:** 10.1128/msystems.00999-24

**Published:** 2024-08-27

**Authors:** Xiaowei Zheng, Li Huang

**Affiliations:** 1 State Key Laboratory of Microbial Resources, Institute of Microbiology, Chinese Academy of Sciences, Beijing, China; 2 College of Life Sciences, University of Chinese Academy of Sciences, Beijing, China; Monash University, Melbourne, Victoria, Australia

**Keywords:** *Hydrogenedentota*, electron bifurcation, [FeFe]-hydrogenase, gene transfer and loss

## Abstract

*Hydrogenedentota*, a globally distributed bacterial phylum-level lineage, is poorly understood. Here, we established a comprehensive genomic catalog of *Hydrogenedentota*, including a total of seven clades (or families) with 179 genomes, and explored the metabolic potential and evolutionary history of these organisms. We show that a single genome, especially those belonging to Clade 6, often encodes multiple hydrogenases with genomes in Clade 2, which rarely encode hydrogenases being the exception. Notably, most members of *Hydrogenedentota* contain a group A3 [FeFe]-hydrogenase (BfuABC) with a non-canonical electron bifurcation mechanism, in addition to substrate-level phosphorylation and electron transport-linked phosphorylation pathways, in energy conservation. Furthermore, we show that BfuABC from *Hydrogenedentota* fall into five sub-types. Phylogenetic analysis reveals five independent routes for the evolution of BfuABC homologs in *Hydrogenedentota*. We speculate that the five sub-types of BfuABC might be acquired from *Bacillota* (synonym *Firmicutes*) through separate horizontal gene transfer events. These data shed light on the diversity and evolution of bifurcating [FeFe]-hydrogenases and provide insight into the strategy of *Hydrogenedentota* to adapt to survival in various habitats.

**IMPORTANCE:**

The phylum *Hydrogenedentota* is widely distributed in various environments. However, their physiology, ecology, and evolutionary history remain unknown, primarily due to the limited availability of the genomes and the lack of cultured representatives of the phylum. Our results have increased the knowledge of the genetic and metabolic diversity of these organisms and shed light on their diverse energy conservation strategies, especially those involving electron bifurcation with a non-canonical mechanism, which are likely responsible for their wide distribution. Besides, the organization and phylogenetic relationships of gene clusters coding for BfuABC in *Hydrogenedentota* provide valuable clues to the evolutionary history of group A3 electron bifurcating [FeFe]-hydrogenases.

## INTRODUCTION

*Hydrogenedentota* bacteria were first found in a clone library of 16S rRNA genes prepared from sediment samples collected in Nankai Trough at a depth of 3,843 m and identified as candidate division NKB19 in 1999 (1). After its genomes were sequenced by using single-cell technology, NKB19 was named *Hydrogenedentes* (2), probably due to its suspected ability to consume or produce hydrogen through [Ni-Fe] hydrogenase, and this name was later amended to *Hydrogenedentota* according to Genome Taxonomy Database (GTDB) release86 for orthography (3, 4). Members of this phylum have been detected in various habitats, such as wastewater, agricultural soil, hot spring, desert, sea water, and polar ice shelf (5–10). However, very little is currently known about this phylum, since no cultured isolates are available, and none of the public genomes of this phylum have been analyzed in detail until now.

Hydrogenases are highly complex metalloenzymes that catalyze the reversible interconversion of H_2_ to 2H^+^ + 2e^−^, enhancing the host’s survival in diverse habitats by utilizing H_2_ as a source of low potential electrons or dispensing excess reducing equivalents to control the cellular redox balance (11, 12). Three evolutionarily distinct types of hydrogenases are classified on the basis of both sequence similarities and compositions of metal-containing catalytic centers and are termed [FeFe]-, [NiFe]-, and [Fe]-hydrogenases (13–17). Whereas [Fe]-hydrogenase is only found in archaea (16), the other two types of hydrogenases are more widely distributed in nature (13, 15, 18, 19). [NiFe]-hydrogenase exists in both bacteria and archaea. [FeFe]-hydrogenase was once believed to be only present in bacteria and eukaryotes. Recently, Greening et al. revealed the presence of [FeFe]-hydrogenase in archaea (20), challenging the previous view that [FeFe]-hydrogenase evolved later in bacteria than [NiFe]-hydrogenase in the last universal common ancestor (15, 21). Previously, [NiFe]-hydrogenase has been annotated in four single-amplified genomes (SAGs) of *Hydrogenedentota* (2), but [FeFe]-hydrogenase has never been reported before in this phylum.

In this study, we first analyzed the global distribution of *Hydrogenedentota* based on 16S rRNA amplicon data from the Earth Microbiome Project (EMP) and then retrieved published metagenome-assembled genomes (MAGs) and SAGs for the genetic and metabolic investigation of the phylum. We show that members of this phylum are globally distributed, employ diverse strategies in energy conservation, and are capable of using a non-canonical catalytic mechanism involving diverse electron bifurcating [FeFe]-hydrogenase (BfuABC) for energy conservation or disposing of excess reducing equivalents. We also reveal multiple evolutionary origins of BfuABC homologs in *Hydrogenedentota*. Our data shed light on the evolution of this non-canonical electron bifurcating [FeFe]-hydrogenases and the strategy of energy conservation of these widespread low-abundance microorganisms in adaptation to surviving in various habitats.

## RESULTS AND DISCUSSION

### The phylum of *Hydrogenedentota* is a globally distributed low-abundance lineage

We retrieved a total of 23,323 qualified samples from the EMP (22). About 35% (8,009) of the samples were found to contain species belonging to *Hydrogenedentota*. These species are distributed around the globe and generally in low abundance in various habitats. They have been found in terrestrial soil, freshwater sediment, marine sediment, fresh water, marine water, and host-associated and other habitats, with the median relative abundances of only 0.013%, 0.054%, 0.067%, 0.006%, 0.002%, 0.019%, and 0.015%, respectively (Fig. S1 and S2; Data Set S1, Sheet 1).

### *Hydrogenedentota* are facultative anaerobic bacteria with a heterotrophic lifestyle

**Fig 1:**
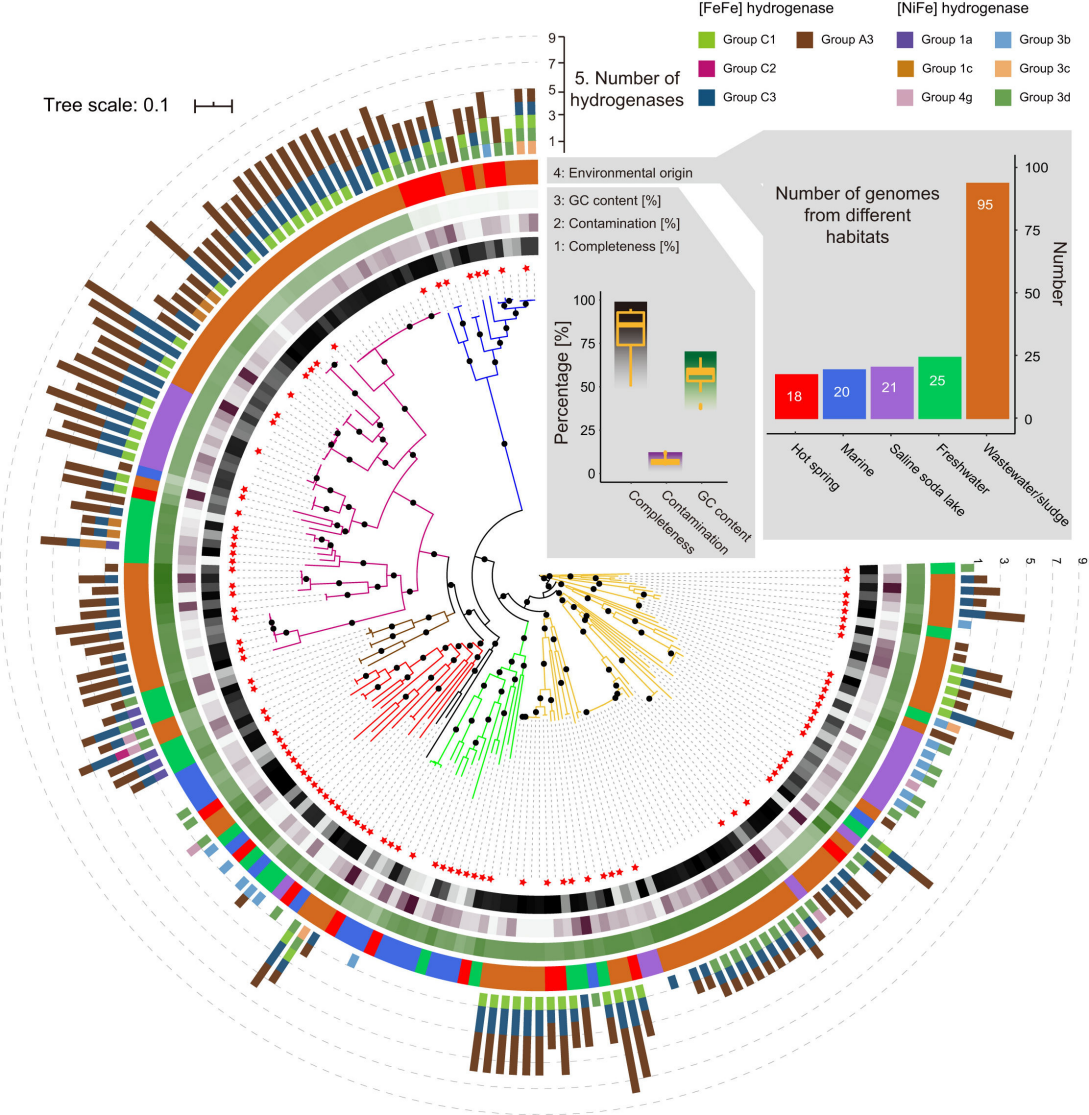
Phylogenomic tree of *Hydrogenedentota* inferred from 120 concatenated marker proteins. Tree annotations from inside to outside circles are (1) completeness (%), (2) contamination (%), (3) GC content (%), (4) environmental origin of the 179 *Hydrogenedentota* genomes, and (5) number of hydrogenases detected in each genome. One hundred non-redundant genomes are indicated by red stars. Branches in yellow, green, black, red, brown, pink, and blue represent seven family-level clades (Clades 1–7, respectively) of *Hydrogenedentota*. The bootstraps are labelled with black dots in the middle of branches when ≥90%. The tree scale bar indicates the mean number of substitutions per site. The bar plot (top right) shows the number of genomes retrieved per environmental category. The box plot (top center) indicates the statistics of completeness, contamination, and GC content estimates of the 179 *Hydrogenedentota* genomes. Shaded gradients are consistent with the values in the heatmap boxes in the tree. Details of the 179 *Hydrogenedentota* genomes are shown in Data Set S1 (Sheet 2).

A total of 179 medium- to high-quality *Hydrogenedentota* genomes (≥50% completeness and ≤10% contamination) were retrieved from NCBI, GTDB, Genomes from Earth’s Microbiomes (GEM), Figshare, and European Nucleotide Archive (ENA) databases (Data Set S1, Sheet 2). These genomes range from 2.68 to 12.10 Mb in size with a median size of 4.68 Mb, and their GC contents were between 36.63% and 69.02% with a median of 59.34% (Fig. 1). The large variation in estimated genome size and GC content points to considerable genome diversity within the phylum. These genomes are grouped into 100 potential strains and 90 candidate species-level taxa, respectively. As revealed by phylogenetic analysis based on 120 concatenated single-copy marker proteins, the 179 genomes are grouped into seven clades (Clades 1–7, Fig. 1; Fig. S3), which may represent seven families in the order of *Hydrogenedentiales* according to the GTDB classification.

**Fig 2:**
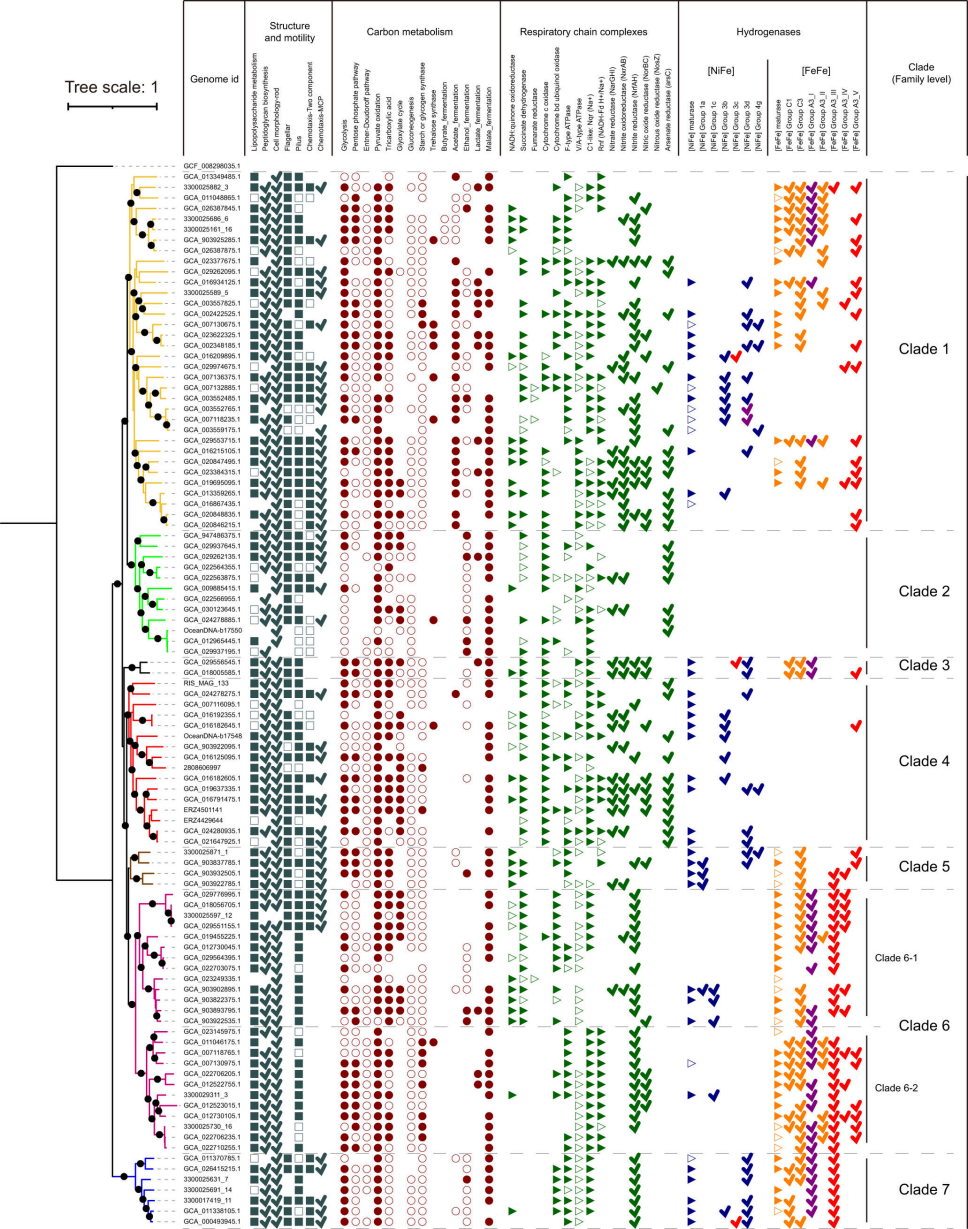
Structure, motility, and metabolic potential of *Hydrogenedentota*. The phylogenomic tree of 100 non-redundant genomes was constructed and annotated as described in Fig. 1. A metabolic pathway or protein complex is indicated with a solid symbol if complete or intact, open symbol if incomplete or partial, and no symbol if absent. A tick indicates the presence of a specific protein or subunit. Those hydrogenases with defined and uncertain electron bifurcating activities are marked by red and purple ticks, respectively. Details of the structure, motility, and metabolic potential of *Hydrogenedentota* can be found in Text S1.

As illustrated in Fig. 2 and Data Set S1 (Sheet 3), members of *Hydrogenedentota* are predicted to have a Gram-negative cell wall, since the Raetz pathway for lipopolysaccharide biosynthesis was identified in over 94% of genomes, and UDP-*N*-acetylmuramoyl-L-alanyl-D-glutamate:*meso*-2,6-diaminopimelate ligase, which is involved in peptidoglycan biosynthesis by adding *meso*-diaminopimelic acid (*m*DAP), the hallmark amino acid residue of Gram-negative bacteria (23), to the pentapeptide, were detected in over 85% genomes. The presence of genes encoding proteins involved in cell division and a rod-shaped cell determination, such as *rodA*, *mreB*, *mreC,* and *mreD* (24, 25), in 97 of the 100 non-redundant genomes suggests that the cells of *Hydrogenedentota* may be rod-shaped.

Further function annotation and interpretation suggest members of *Hydrogenedentota* are motile bacteria with a heterotrophic lifestyle (Fig. 2; Data Set S1, Sheets 3–5; see Text S1 for details). *Hydrogenedentota* appear to be capable of generating ATP via substrate-level phosphorylation (SLP) since genes encoding pyruvate kinase and phosphoglycerate kinase, two key enzymes involved in the phosphor group transfer from phosphoenolpyruvate or 1,3-diphosphoglycerate to ADP during glycolysis, were annotated in over 68% (123) and 83% (150), respectively, of the 179 genomes (Data Set S1, Sheet 4).

To gain insights into the energy conservation of *Hydrogenedentota*, we also characterized the electron transport chain (ETC) of the organisms. As shown in Fig. 2 and Data Set S1 (Sheet 4), complex I (NADH-quinone oxidoreductase) appears to be patchily distributed across the seven clades of *Hydrogenedentota* and found in only 36% of the genomes. However, electrons may be fed into ETC in genomes lacking complex I by complex II (succinate dehydrogenase) or Na^+^ translocating NADH:quinone oxidoreductase (Na^+^-NQR). Other quinone reductases, such as glycerol-3-phosphate dehydrogenase and quinoprotein glucose dehydrogenase, which are involved in glycerophospholipid and carbohydrate metabolism, respectively, are found in almost half of the members of *Hydrogenedentota*. These observations suggest the presence of diverse pathways for electrons entering the respiratory chain in *Hydrogenedentota*. While both complex IV, caa3-type heme–copper oxygen reductase and cytochrome bd oxidase, were detected, nearly 70% of the genomes have at least one of them, indicating that *Hydrogenedentota* mainly use O_2_ as the electron acceptor. However, oxygen reductase may also serve to protect oxygen-sensitive enzymes in strictly anaerobic organisms when they are exposed to oxic conditions, as shown in *Desulfovibrio desulfuricans* ATCC 27774 (26). It is worth noting that ubiquinone (UQ or coenzyme Q), usually used by aerobic organisms, is speculated to have appeared about 2.5 billion years ago as a strategy of life to adapt to rising oxygen levels (27, 28), whereas menaquinone (MK or vitamin K2) exists in microorganisms living under low O_2_ or anoxic conditions (29). *Hydrogenedentota* possess a complete set of men-like genes for the synthesis of MK, while genes encoding UQ synthesis are incomplete in this phylum (Data Set S1, Sheets 4 and 5). In addition, genes encoding nitrite reductase, nitrate reductase and nitric oxide reductases were detected, suggesting that the organisms are capable of using NO_2_^−^, NO_3_^−^, or NO as electron acceptors. Further, over half (95) of the 179 *Hydrogenedentota* genomes are derived from anaerobic environments, such as wastewater, sludge, and bioreactor (Data Set S1, Sheet 2). These results are consistent with the notion that *Hydrogenedentota* are facultative anaerobes. We also found that FoF1-ATPase and V/A-ATPase are encoded in 61% and 71% of the *Hydrogenedentota* genomes, respectively (Fig. 2; Data Set S1, Sheet 4). The co-existence of the two types of ATP synthases may offer fitness advantages for survival in various habitats (30–33). Taken together, our data indicate that *Hydrogenedentota* primarily employ electron transport-linked phosphorylation (ETP) in energy conservation.

Strikingly, these organisms have evolved an electron bifurcation pathway, in addition to SLP and ETP. Specifically, group A3 [FeFe]-hydrogenases (Fig. 2; Fig. S4; Data Set S1, Sheet 6), which use either H_2_ as a source of reducing power or H^+^ as an oxidant to dispose of excess reducing equivalents (13, 34), are more widely distributed than [NiFe]-hydrogenases in *Hydrogenedentota*. This contrasts sharply with the previous belief (2). We identified six types of [NiFe]-hydrogenases (groups 1a, 1c, 3b, 3c, 3d, and 4g) in *Hydrogenedentota* (Fig. 1 and 2; Fig. S4e; see Text S1 for details), among which only group 3c [NiFe] methyl viologen-reducing (Mvh) hydrogenases are believed to have electron bifurcating activity (35). Due to the lack of two FeS clusters in the hoxF/HydB subunit, a homolog of diaphorase (36) or the BfuB subunit in group A3 [FeFe]-hydrogenase (35, 37) (see below), group 3d [NiFe]-hydrogenases from *Hydrogenedentota* are probably incapable of electron bifurcation (Fig. S4f). The reversible process catalyzed by the hydrogenases is referred to as electron bifurcation in this report for convenience. Notably, members of *Hydrogenedentota* are often found to encode either [FeFe]- or [NiFe]-hydrogenase, and the two types of hydrogenases are rarely encoded in the same genome (Fig. 1 and 2).

### Electron bifurcating [FeFe]-hydrogenases have diversified into distinct sub-types in *Hydrogenedentota*

Four types (492 sequences) of putative [FeFe]-hydrogenases, i.e., groups C1 (53), C2 (1), C3 (156), and A3 (282), were identified in the 179 *Hydrogenedentota* genomes (Fig. 2; Fig. S4a; Data Set S1, Sheet 6). Group C [FeFe]-hydrogenases are believed to be involved in hydrogen sensing (15). However, we were hardly able to find the H-cluster (i.e., the hydrogenase active site consisting of a [4Fe4S] cluster bound via a cysteine to a 2Fe sub-cluster) binding motifs P1, P2, and P3 in group C1 [FeFe]-hydrogenases from *Hydrogenedentota* by using the reported signature sequences (14, 17, 38). Therefore, it is unclear if they are capable of sensing hydrogen. Twenty-one of the 53 genes encoding group C1 [FeFe]-hydrogenases and nearly all the genes encoding group C3 [FeFe]-hydrogenases are located next to those encoding group A3 [FeFe]-hydrogenases (Fig. S4c and S5). This finding indicates that group C [FeFe]-hydrogenases from *Hydrogenedentota* are mainly involved in the regulation of the production and consumption of hydrogen. It is difficult to speculate the physiological processes in which the other 32 group C1 [FeFe]-hydrogenases are involved, based on the analysis of genes adjacent to their coding genes due to the limitation incurred by the length of contigs or scaffolds (Data Set S1, Sheet 11). Besides, a histidine kinase/HSP90-like ATPase (*HATPase_C*) domain, often found in ATP-binding proteins, e.g., histidine kinase (39) and topoisomerases (40), was detected in group C1 [FeFe]-hydrogenases from *Hydrogenedentota* (Fig. S4d). However, this domain is absent in the best-matched group C1 [FeFe]-hydrogenase in the HydDB database (Fig. S4d) (41). Unlike group C1, two types of C3 [FeFe]-hydrogenases (corresponding to the T subunit in sub-types IV and V of BfuABC, respectively, see below) contain a PAS domain (42) and a histidine kinase domain (*His_kinase_dom*), respectively, which play a regulatory role. These two domains are also found in the best hits of two types of C3 [FeFe]-hydrogenases in the HydDB database (WP_005996231.1 and WP_006928132.1) (41) (Fig. S4d and S3f).

Since group C2 [FeFe]-hydrogenase was found only in a single genome, and genes encoding groups C3 and A3 [FeFe]-hydrogenases are usually located together in the same gene cluster (Fig. S5), we will focus our analysis on group A3 [FeFe]-hydrogenase and its encoding gene cluster. Three phylogenetically distinct groups of flavin-based electron bifurcating (FBEB) enzymes, i.e., EtfAB, NfnAB, and Hdr-Mvh complexes, are known to use FAD to bifurcate electrons (43–45). However, BfuABC is recently proposed to use a different electron bifurcation mechanism involving the combination of a single FMN and nearby iron sulfur clusters (35, 37). This non-canonical electron bifurcation mechanism suggests that BfuABC might not be FBEB enzymes and represent a novel type of electron bifurcating enzymes, which, unlike flavin (only)- and quinone-based electron bifurcating enzymes, use a unique “FMN-FeS” center to bifurcate electrons (35).

**Fig 3:**
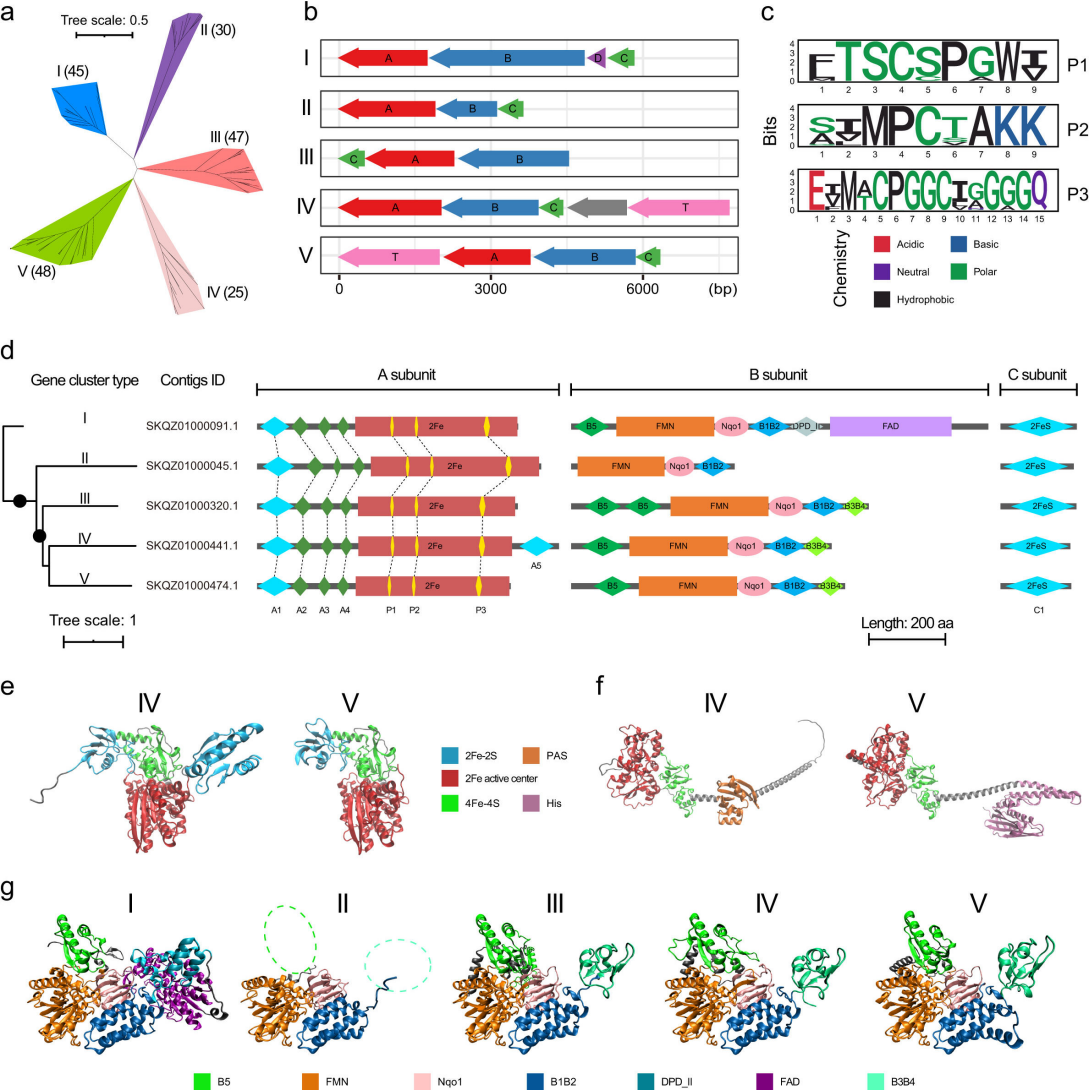
Phylogenetic characteristics of group A3 [FeFe]-hydrogenases (BfuABC) in *Hydrogenedentota*. (a) Unrooted radiation tree based on the concatenate of three subunits “ABC” from 195 nearly complete group A3 BfuABC [FeFe]-hydrogenases. Numbers in parentheses represent the number of hydrogenases in corresponding sub-types. (b) Five sub-types of gene clusters for group A3 [FeFe]-hydrogenases in *Hydrogenedentota*. To facilitate comparison, the gene orders are displayed according to the gene clusters located on the negative (−) DNA strand. (c) Schematic diagram indicating the extent of conservation in residues comprising the H-cluster motifs (P1, P2, and P3) in *Hydrogenedentota* BfuA. Residues with higher bit scores (i.e., larger font sizes) represent a greater degree of conservation at a given aligned position. The property of a given amino acid is indicated by a color. (d) Unrooted phylogenetic tree of the concatenate of three subunits “ABC” and domains of BfuB subunit of group A3 [FeFe]-hydrogenase in CSSed162cmA_463 (GCA_007118765.1). (e) Ribbon diagram showing the predicted structures of sub-type IV and V BfuA subunits. (f) Ribbon diagram showing the predicted structures of sub-type IV and V BfuT subunits (group C3 [FeFe]-hydrogenase). “PAS” represents a signal sensor domain, named after three proteins that it occurs in: Per-period circadian protein, Arnt-Ah receptor nuclear translocator protein, and Sim-single-minded protein. “His” represents a histidine kinase domain (*His_kinase_dom*). (g) Ribbon diagram showing the predicted structures of five sub-types BfuB. The two dotted coiled regions in sub-type II represent the missing binding domains of the “B5” and “B3B4” (the cause of the lack of electron bifurcating) FeS clusters, respectively. In panels (d and e), “A1,” “A5,” “B2,” “B5,” and “C1” represent [2Fe-2S] clusters; “A2,” “A3,” “A4,” “B1,” “B3,” and “B4” represent [4Fe-4S] clusters; “2Fe” represents the active center for hydrogen production or consumption; H-cluster should contain a “2Fe” center and a [4Fe-4S] cluster. “FMN,” flavin mononucleotide binding domain; “Nqo1,” soluble ligand binding β-grasp (SLBB) domain within a region of Nqo1 middle domain-like superfamily; “DPD_II,” dihydroprymidine dehydrogenase domain (DPD_II) carrying two [4Fe-4S] clusters; “FAD,” flavin adenine dinucleotide (FAD) or NAD(P) binding domain. The detailed characteristics of all of the 195 gene clusters and BfuB subunit domains are shown in Fig. 6 and S5. The genome of CSSed162cmA_463 (GCA_007118765.1), which contains all five types of gene clusters, is shown here as an example to illustrate the features of gene clusters and BfuA, BfuB, and BfuT sub-units’ domains of group A3 [FeFe]-hydrogenases in *Hydrogenedentota*. A gene, denoted T (in sub-type IV gene cluster), in the genome of CSSed162cmA_463 (GCA_007118765.1) is a partial gene residing on the edge of the contig (SKQZ01000441.1; Fig. S5d). SKQZ01000441.1 is nearly 100% identical to SLMA01000044.1 in the genome of CSSed165cm_333 (GCA_007133815.1). The average nucleotide identity value of the two genomes is close to 100%, indicating that they belong to the same species (Fig. S3). Therefore, we speculate that the partial T gene may have resulted from incomplete assembly, and the T gene is thus shown in CSSed162cmA_463 (GCA_007118765.1).

To understand the diversity of BfuABC in *Hydrogenedentota* and predict the roles of the three subunits in hydrogen production/consumption (BfuA) and bifurcation (BfuB and BfuC) (13, 14, 34, 37, 46), we looked into their organizational features. Gene clusters encoding no or an incomplete BfuB or BfuC subunit in the neighborhood (i.e., within five genes up- or downstream) of the *bfuA* gene were not retained for further analysis. A total of 195 gene clusters (Fig. S5) were predicted to encode an intact trimeric BfuABC or tetrameric BfuABCD complex. As shown in Fig. 3a and Fig. S6, both the individual and concatenated phylogeny analyses of BfuA, BfuB, and BfuC reveal five independent clusters (termed sub-types I to V). Interestingly, the five BfuABC sub-types nearly perfectly correspond to five distinct patterns of gene cluster organization (Fig. 3b; Fig. S5). To the best of our knowledge, the pattern of BfuABC encoded in the “BAC” orientation, as shown in sub-type III, has never been reported.

BfuA, which is involved in ligating the H-cluster, contains three motifs, i.e.*,* P1 (xTSCxPxWx), P2 (xxMPCxAKK), and P3 (ExMxCPGGCxxGxGQ). Although these motifs are in general conserved, several positions in each of the three motifs vary significantly among the five sub-types (Fig. 3c; Fig. S7). For example, Cys at the fifth position in P1 is often present in sub-type IV while Ser is common in the other four sub-types. Only BfuA in sub-type IV exhibits the “M3c” modular structure (14), which is predicted to contain three [4Fe-4S] clusters (“A2”, “A3,” and “A4”) and two [2Fe-2S] clusters (“A1” and “A5”), of which “A5” is located at the C-terminal end of the protein (Fig. 3d and e). A “M3a” modular structure is typical of sub-types I, II, III, and V. The variations at the motif and domain levels suggest the evolutionary independence of BfuAs of the five sub-types. However, because of the lack of biochemical validation, it remains uncertain whether, or to what extent, these changes affect the oxidation or production of hydrogen.

The presence and location of FeS clusters in BfuB homologs have been verified in HydABC (BfuABC) [FeFe]-hydrogenase from *Thermotoga maritima* and a related HydABC hydrogenase (NiFe-HydABCSL) from *Acetomicrobium mobile* DSM 13181 by cryo-electron microscopy (35, 37, 46). A complete set of “B1” to “B5” FeS clusters appears to be necessary for the activity of bifurcating hydrogenases. In support of this notion, this set is incomplete in non-bifurcating homologous Fdh (formate dehydrogenase) and Nqo (NADH quinone oxidoreductase, complex I) enzymes (35, 47). In addition, a HydABC (BfuABC) [FeFe]-hydrogenase from the *Syntrophomonas wolfei* DSM 2245B, whose HydB (BfuB) subunit lacks “B3” and “B4” FeS clusters, was shown to lack the bifurcating ability in *in vitro* assays (48, 49). Interestingly, the non-bifurcating hydrogenase (HydABC) was still able to produce hydrogen from NADH without the need for Fd at low hydrogen partial pressure (48, 49). Thus, it is suggested that “B1” and “B2” FeS clusters are capable of reversible electron transfer between NADH and protons, and “B3” and “B4” FeS clusters play a role in interacting with Fd. Similarly, we propose the following complete and reversible electron transport pathways for [FeFe]-hydrogenases from *Hydrogenedentota*. Two electrons generated from H_2_ by the H-cluster domain in BfuA are transferred to FMN via the “B1” FeS cluster in the electron bifurcating BfuBC core, followed by bifurcation to the first electron acceptor NAD (high potential) directly and the second electron acceptor Fd (low potential) through FeS clusters in the order of “C1-B2-B3-B4”.

Some suggest that “B5” FeS clusters may display the bifurcating ability since the “B5” and “C1” FeS clusters can act as a gate or switch separating the low potential electron pathway (“B2-B3-B4”) from the mid potential electron pathway (“FMN-B1”) (35), while others argue that the “B5” FeS cluster is at a dead end and unlikely involved in electron transfer (37). Therefore, the function of double “B5” FeS clusters in sub-type III BfuB is still unclear. Interestingly, the domain containing the “B3” and “B4” FeS clusters is replaced by a dihydropyrimidine dehydrogenase domain (DPD_II) with an extra FAD/NAD(P)-binding domain located at the C-terminus of the protein in sub-type I BfuB (Fig. 3d and g). This potentially creates an alternative bifurcation center of “FAD” in addition to the “FMN-FeS” center. As aforementioned, FAD is able to bifurcate electrons alone without the need for FeS clusters in a process known as a canonical bifurcating pathway, as found in FBEB enzymes (43–45). However, the co-existence of “FAD” and “FMN-FeS” bifurcating centers in a single BfuABC hydrogenase has never been reported. The extra FAD binding domain would induce conformational changes in the enzyme, possibly altering both flavin redox properties and electron transport. However, more studies are required to determine if both, either, or neither of the two potential bifurcation centers bifurcate electrons. We postulate that sub-type I BfuABC may represent a novel bifurcating mechanism as it possesses a possible bifurcating “FAD” center in addition to the “FMN-FeS” center.

### The gene cluster coding for BfuABC is highly mobile among bacterial phyla

**Fig 4:**
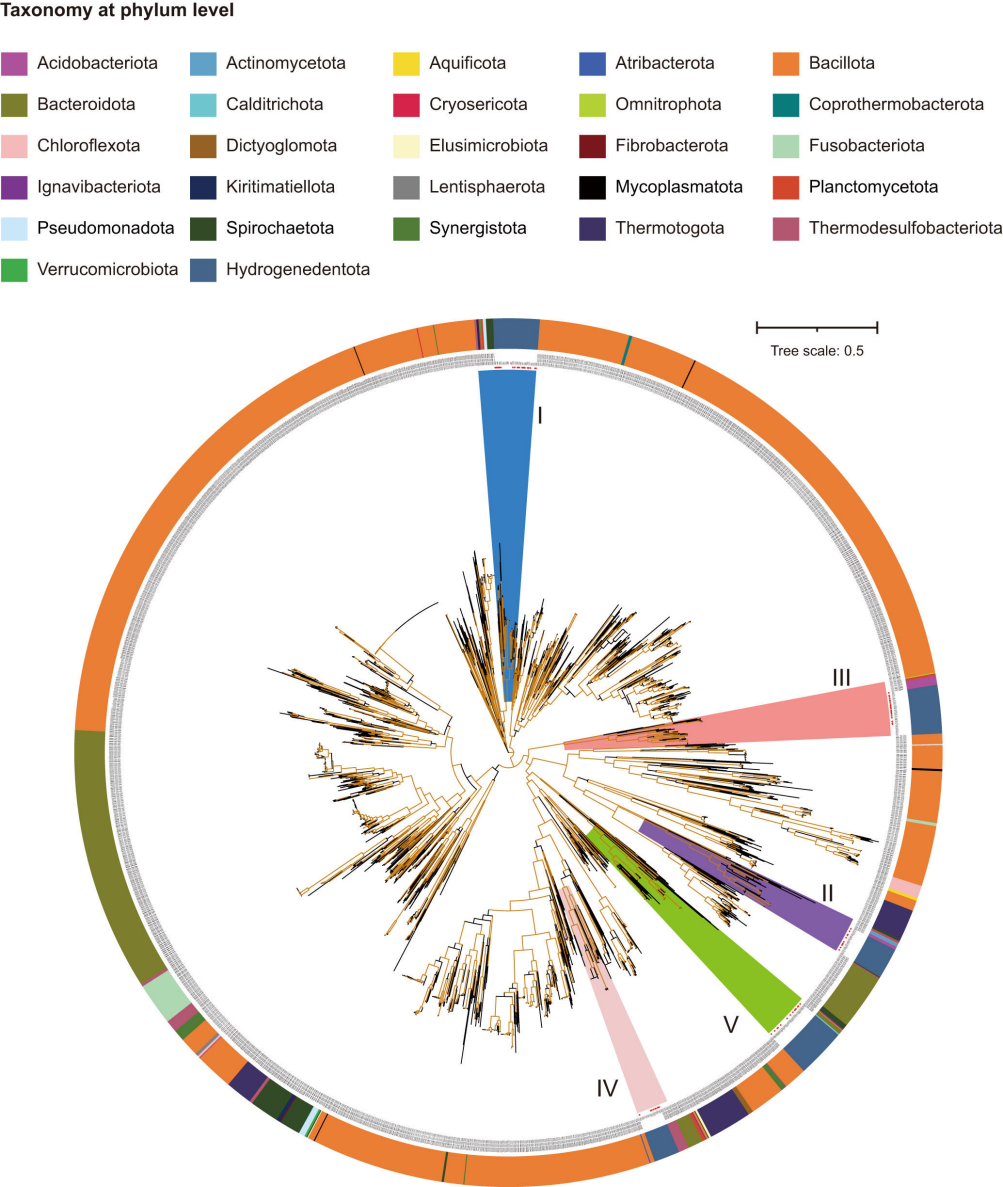
Phylogenetic analysis of *Hydrogenedentota* BfuABC proteins in relation to those in other bacterial phyla. The strip with different colors in the outer ring indicates different bacterial phyla. Sub-types I to V of BfuABC are marked in blue, purple, red, pink, and green, respectively. The bootstraps of over 90% are shown in brown branches. The genome id, gene cluster types of BfuABC homologs, and domains of BfuB subunit are displayed sequentially at the end of the branches. “X” represents genes that encode subunits or proteins other than BfuABCDT. The gene orders and orientations and BfuB domains are marked as in Fig. 3.

To interrogate the evolutional relationship of the five sub-types of BfuABC [FeFe]-hydrogenases from *Hydrogenedentota*, we aligned the 195 BfuABC concatenated sequences against a local reference genome database and selected a total of 2,415 BfuABC homologs from 2,144 reference genomes from species belonging to 26 bacterial phyla (Fig. 4; Data Set S1, Sheet 7). The phylogenetic tree of the 2,415 BfuABC homologs from reference genomes, together with 195 BfuABC homologs from *Hydrogenedentota* genomes, point to complex evolution pathways for group A3 [FeFe]-hydrogenases (Fig. 4). As shown in Fig. 4, there are substantially more BfuABC [FeFe]-hydrogenases than just the five sub-types identified in *Hydrogenedentota*. Phylum *Bacillota* (synonym *Firmicutes*) contains the most diverse group A3 [FeFe]-hydrogenases. Intriguingly, group A3 [FeFe]-hydrogenases do not appear to be strictly vertically inherited, as revealed by phylogenetic and taxonomic conflicts both at and below the phylum level (Fig. 4). For example, the BfuABC homologs from *Bacillota* are not all clustered together but are scattered, forming clusters with those from other 26 bacterial phyla. These results suggest that inter-phylum horizontal gene transfer has been a major driving force in the evolution of group A3 [FeFe]-hydrogenases.

**Fig 5:**
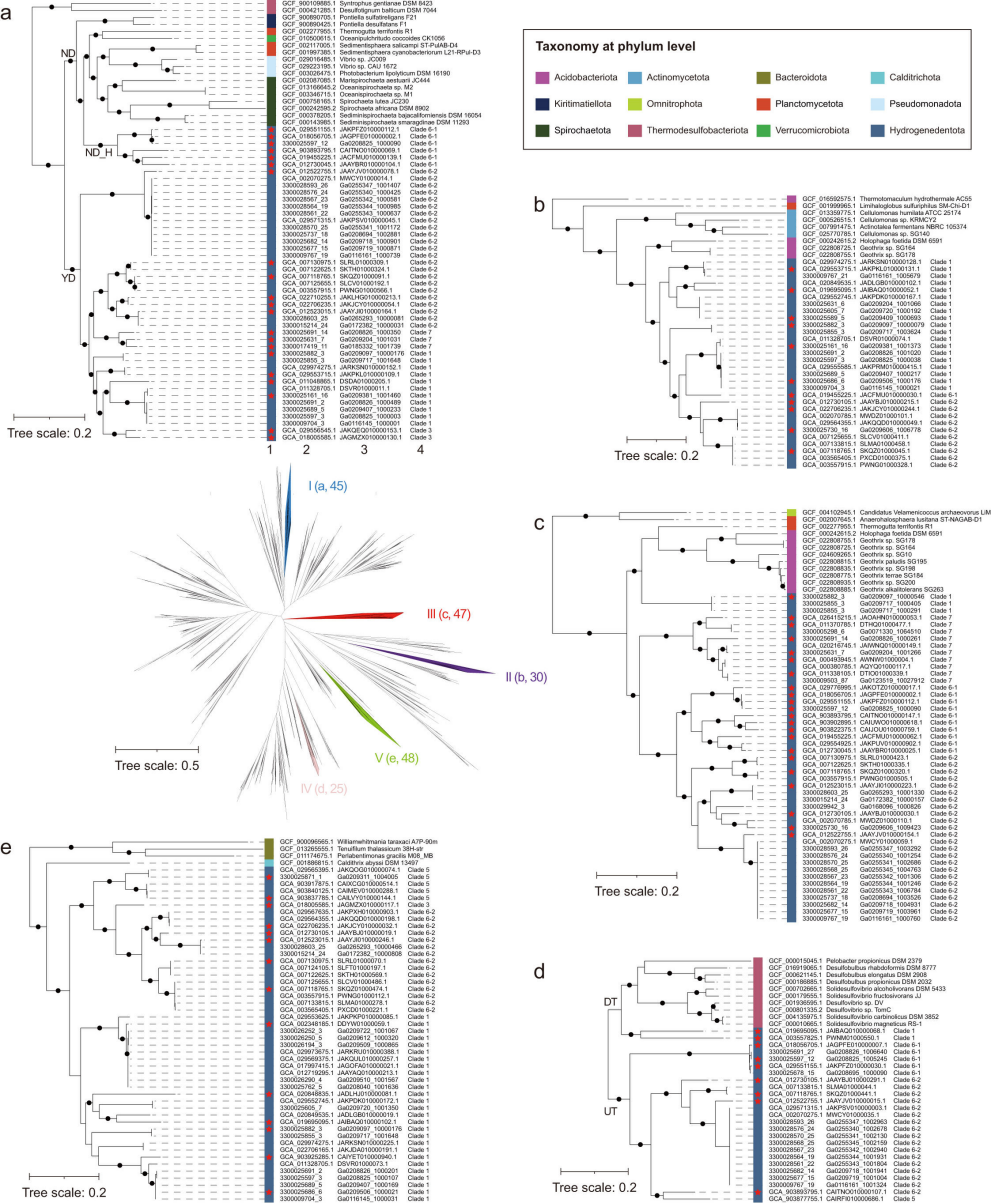
Phylogenetic analysis of five sub-types of BfuABC proteins in *Hydrogenedentota* and other bacterial phyla. The radiation tree was constructed based on the concatenate of the three subunits “ABC” of 195 and 2,144 BfuABC homologs from the genomes of *Hydrogenedentota* and the other 26 bacterial phyla, respectively. Sub-types I to V of BfuABC are marked in blue, purple, red, pink, and green, respectively, and numbers in parentheses represent the number of hydrogenases in corresponding sub-types in the radiation tree. More information, shown in a circular form of this tree, is available in Fig. 4. Panels (a–e) are enlarged regions I to V of the radiation tree, respectively, and show the detailed relationships between the sub-types I to V of BfuABC in *Hydrogenedentota* and those in other closely related phyla. “1,” phylum affiliation; “2,” genome id; “3,” contig id or strain name in other phyla; “4,” clade in *Hydrogenedentota*. Clades 6-1 and 6-2 are two sub-clades of Clade 6, as illustrated in Fig. 2; Fig. S3. Nodes “ND” and “YD” in panel (a) represent homologs in the corresponding clusters with and without a BfuD subunit, respectively, and “H” in “ND_H” means “*Hydrogenedentota.*” Nodes “UT” and “DT” in panel (e) represent the location of *bfuT* either upstream or downstream of *bfuA* in the gene clusters. The non-redundant *Hydrogenedentota* genomes at strain levels are indicated by red stars. The bootstraps are labeled with black dots when ≥90%.

Moreover, the five sub-types appear to be distributed patchily among *Hydrogenedentota*. Although this may be attributed in part to the incompleteness of the genome sequences, it more likely reflects the results of gene transfer and loss, two key drivers of gene changes in the evolution of *Hydrogenedentota*, as revealed by amalgamated likelihood estimation (ALE) (Fig. S8; Data Set S1, Sheet 10). To shed more light on the evolution of electron bifurcating [FeFe]-hydrogenases from *Hydrogenedentota*, we then examined the individual phylogenetic clusters containing the five sub-types of BfuABC homologs and their nearest neighbors. As shown in Fig. 5, the five BfuABC sub-types appear to have been initially acquired from *Bacillota* through separate horizontal gene transfer events, and thus, each of them exhibits unique evolutionary features.

Sub-type I BfuABC homologs from *Spirochaetota*, *Pseudomonadota*, *Planctomycetota*, *Verrucomicrobiota,* and *Kiritimatiellota,* which are most closely related to that from *Hydrogenedentota*, generally lack BfuD (node ND in Fig. 5a). Unexpectedly, several homologs from *Hydrogenedentota* in Clade 6 also lack BfuD (node ND_H in Fig. 5a; Fig. S5). Interestingly, BfuD is present in a distantly related homolog from *Syntrophus gentianae* DSM 8423 and absent in a homolog from *Desulfotignum balticum* DSM 7044. Both of the strains belong to phylum *Thermodesulfobacteriota*, suggesting that BfuD might be susceptible to loss during evolution. Similarly, as shown in Fig. 4 and 5c, the ancestral sub-type III BfuABC homolog might contain a BfuD subunit and subsequently lost it in *Hydrogenedentota* and some lineages in *Acidobacteriota* and *Planctomycetota* (*Thermogutta terrifontis* R1) since this subunit is encoded by both *Planctomycetota* (*Anaerohalosphaera lusitana* ST-NAGAB-D1) and *Ca*. Omnitrophota (*Velamenicoccus archaeovorus* LiM) (Fig. 4 and 5c). All closely related sub-type II BfuABC homologs from *Acidobacteriota*, *Actinomycetota,* and *Planctomycetota* lack the “B3,” “B4,” and “B5” FeS clusters (Fig. 4 and 5b). Both the least and most related species are from the phylum of *Acidobacteriota* (*Thermotomaculum hydrothermale* AC55 and *Holophaga foetida* DSM 6591), suggesting that sub-type II BfuABC homologs in *Hydrogenedentota* and *Acidobacteriota* were inherited from a common ancestor. Horizontal gene transfer of sub-type II *BfuABC* within *Hydrogenedentota* may occur frequently, especially in Clade 1, where the BfuABC homologs are not clustered together (Fig. 5b).

As shown in Fig. 4, sub-types VI and V BfuABC homologs reside in the same large cluster, indicating their close evolutionary relationship. These two sub-types are featured by a *bfuT*, an extra gene encoding a group C3 hydrogen-sensing [FeFe]-hydrogenase. *bfuT* is often located upstream of *bfuC* in a sub-type IV gene cluster, which mainly exists in the genomes of Clade 6 (Fig. 5d). However, two exceptions were observed. First, *bfuT* is downstream of and adjacent to *bfuA* in the two genomes of Clade 1 (GCA_019695095.1 and GCA_003557825.1) (Fig. S5d). All of the most closely related BfuABC homologs of the two genomes are from species in phylum *Thermodesulfobacteriota*. No *bfuT* was found downstream of *bfuA* in these thermodesulfobacteria, except for *Desulfobulbus elongatus* DSM 2908 and *Desulfobulbus propionicus* DSM 2032 (Fig. 4 and 5d). Second, the *bfuT* and *bfuA* genes in the *D. elongatus* DSM 2908 and *D. propionicus* DSM 2032 genomes are separated by three genes (Fig. 4). These observations suggest that BfuABC homologs from *Thermodesulfobacteriota* and *Hydrogenedentota* share a common ancestor, which was acquired through horizontal gene transfer before the divergence of the two phyla, and exhibit separate evolution trajectories, resulting in variation in the location of *bfuT* in relation to *bfuA*. Puzzlingly, one of the two evolutionary paths (node DT in Fig. 5d) appears to have flourished in descendants (Clade 6), while the other (node UT in Fig. 5d) does not in *Hydrogenedentota*. In contrast, sub-type V gene clusters are inherited vertically in both Clades 1 and 6. The organization of a sub-type V gene cluster from *Hydrogenedentota* is consistent with that from phylum *Calditrichota* (Fig. 4 and 5e), represented by *Caldithrix abyssi* DSM 13497. However, three distantly related BfuABC homologs from phylum *Bacteroidota* (*Perlabentimonas gracilis* M08_MB, *Tenuifilum thalassicum* 38H-str, and *Williamwhitmania taraxaci* A7P-90m) have an additional *bfuT* gene upstream of *bfuC* in addition to one downstream of *bfuA*. The physiological and ecological implications of the presence of two *bfuT* genes in a single gene cluster are unclear but may concern habitat adaptation. We speculate that both *Hydrogenedentota* and *Calditrichota* have lost the second *bfuT* after initially obtaining it through ancient horizontal gene transfer from *Bacteroidota*. Clearly, BfuT is also susceptible to gain and loss during the evolution of both sub-type IV and V BfuABC homologs.

### Electron bifurcating [FeFe]-hydrogenases increase the versatility of *Hydrogenedentota* in energy conservation

**Fig 6:**
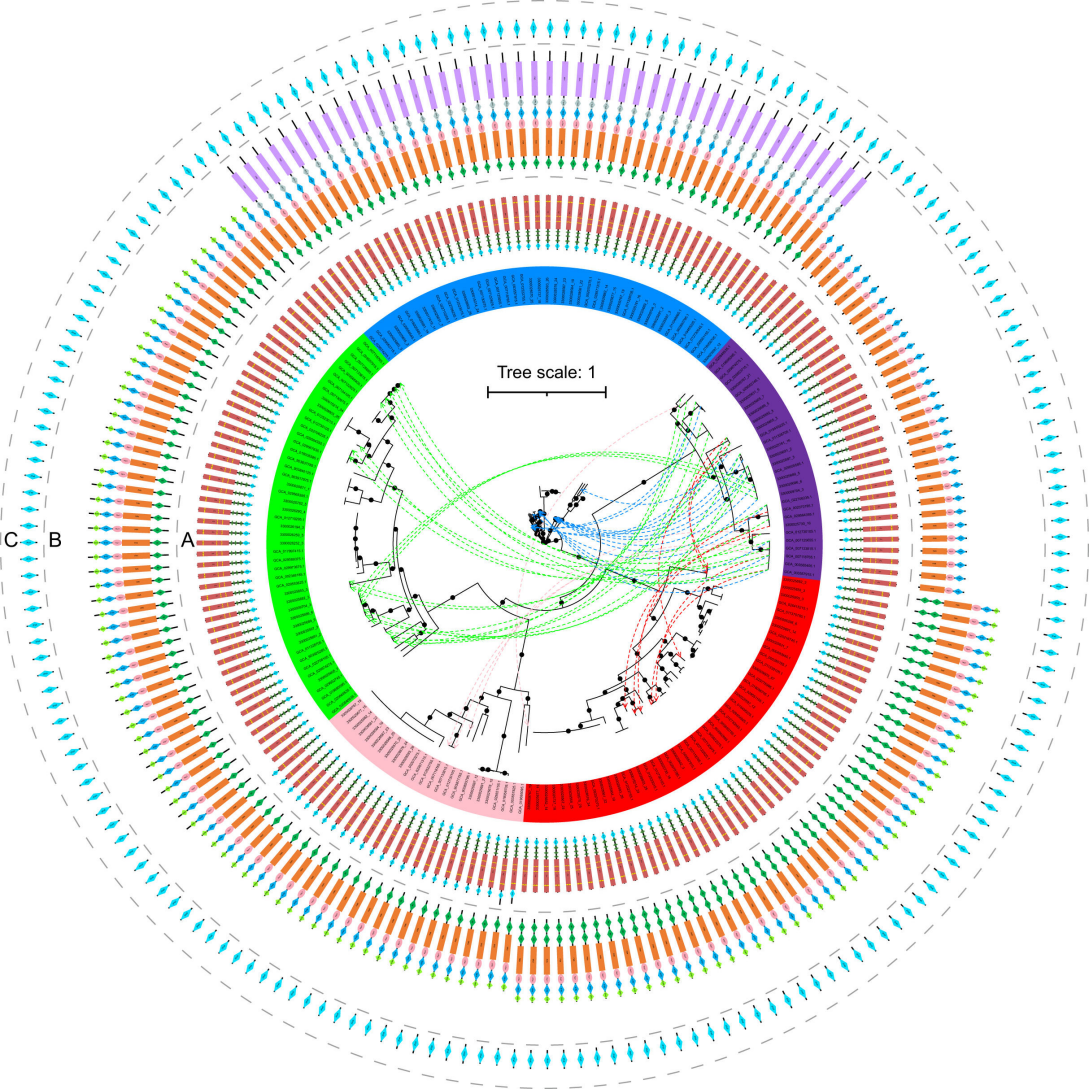
Phylogenetic tree of the concatenate of the three subunits “ABC” from the 195 nearly complete group A3 [FeFe]-hydrogenases in *Hydrogenedentota*. Five sub-types (I to V) of group A3 [FeFe]-hydrogenases, i.e., BfuABDC, BfuABC, BfuCAB, BfuABCT, and BfuTABC, are shown by blue, purple, red, pink, and green strips in genome id, respectively. Domain structures of BfuA, BfuB, and BfuC are shown outside sequentially. For a genome containing the gene cluster coding for BfuABC as well as any of those coding for BfuABDC, BfuCAB, BfuABCT, and BfuTABC, the coexistence of the gene clusters is indicated by dotted lines in color.

Genes encoding bifurcating [FeFe]-hydrogenases of multiple sub-types often co-exist in a single genome. For example, CSSed162cmA_463 (*Hydrogenedentota*, GCA_007118765.1) has all the five sub-types (Fig. 3 and 6), and *Anoxybacter fermentans* DY22613 (*Bacillota*, GCF_003991135.1) has three (Fig. 4). Interestingly, nearly all of the strains containing sub-type II BfuABC complexes, which lack the electron bifurcating ability, have at least one of the other four sub-types of putative bifurcating [FeFe]-hydrogenases (Fig. 6). Moreover, Clade 6-1 and Clade 6-2 genomes are capable of translocating protons or Na^+^ ions across the cytoplasmic membrane using different mechanisms for energy generation through ATP synthase. Clade 6-1 genomes usually possess complexes I (NADH-quinone oxidoreductase), II (succinate dehydrogenase), and IV (cytochrome bd oxidase or cyt-bd oxygen reductase) for transporting electrons successively while translocating protons across the membrane at the same time (Fig. 2). On the other hand, Clade 6-2 genomes encode a Rnf complex, a Na^+^-dependent ferredoxin:NAD^+^ oxidoreductase, instead of the above three respiratory chain complexes. Rnf functions to generate a transmembrane electrochemical Na^+^ gradient by coupling the reduction of NAD^+^ with reduced ferredoxin. Interestingly, all of the genomes lacking the gene encoding BfuD of sub-type I [FeFe]-hydrogenase belong to Clade 6-1 (node ND_H in Fig. 5a), and those having the BfuD-encoding gene belong to Clade 6-2 (node YD; Fig. 5a). Therefore, unlike Clade 6-1, which uses respiratory chain complexes, Clade 6-2 may couple reactions catalyzed by Rnf with those by BfuABCD homologs in generating the transmembrane electrochemical gradient and maintaining intracellular redox balance. And the tetrameric complex of sub-type I BfuABCD may facilitate the oxidation of NADH generated by Rnf more efficiently than the trimeric one due to the presence of BfuD functioning in rapid regulatory response to fluctuating environmental conditions (50). In other words, the interaction between reactions catalyzed by BfuABCD and those by Rnf is beneficial or even vital for *Hydrogenedentota* to obtain an adequate energy supply in adapting to changing environments.

### Conclusions

*Hydrogenedentota* is a group of globally distributed and low-abundance bacteria living in various habitats. Based on the analysis of currently available genomes, members of this phylum are predicted to be Gram-negative, rod-shaped, motile, facultative anaerobes with a heterotrophic lifestyle. Most of these organisms have evolved a non-canonical electron bifurcation pathway, in addition to SLP and ETP, as a strategy for energy conservation. Specifically, they employ the electron bifurcating [FeFe]-hydrogenase (group A3) in hydrogen metabolism, a process presumably vital for their survival in diverse habitats. Five independent pathways have been proposed for the evolution of the gene clusters coding for BfuABC in *Hydrogenedentota*, and these gene clusters are predicted to have been subjected to extensive intra- and interphyla horizontal gene transfers and undergone gene losses and gains.

## MATERIALS AND METHODS

### 16S rRNA gene amplicon data set

Sample processing, sequencing, and core amplicon data analysis were performed by the EMP (www.earthmicrobiome.org), and all amplicon sequence data and metadata are publicly available through the EMP data portal (http://qiita.microbio.me/emp) (22). The global distribution of *Hydrogenedentota* was analyzed as described previously (51).

### *Hydrogenedentota* genome collection

Published MAGs or SAGs of *Hydrogenedentota* (2, 9, 52–54), verified by GTDB-Tk (v2.0.0) (55), were retrieved from the NCBI Assembly database (ftp://ftp.ncbi.nlm.nih.gov/genomes/genbank/bacteria/, July 2023), GTDB (https://data.gtdb.ecogenomic.org/releases/release207/), GEM catalog (https://portal.nersc.gov/GEM) (56), Figshare (https://doi.org/10.6084/m9.figshare.c.5564844.v1) (54), and ENA (https://www.ebi.ac.uk/ena/browser/view/PRJEB45951 and https://www.ebi.ac.uk/ena/browser/view/PRJEB35712) (9, 53). All these MAGs and SAGs were collectively referred to as “genomes” in this article for simplicity.

### Genome completeness assessment, dereplication, and phylogeny

The completeness, potential contamination, and strain heterogeneity of these genomes were evaluated via CheckM (v1.1.9) with lineage-specific marker genes (57). Genome size was estimated as described by Chen et al. (58). One hundred seventy-nine medium- to high-quality (completeness ≥50% and contamination ≤10%) genomes were retained in downstream analysis (Data Set S1, Sheet 2). To reduce redundancy, the 179 genomes were dereplicated at 99% average nucleotide identity using dRep (v2.3.2; option: -comp 50 -con 5 -sa 0.99) (59), resulting in a total of 100 strain-level genomes. Phylogenetic analysis of the above genomes based on 120 bacterial marker proteins were conducted using the “identify” and “align” steps in GTDB-Tk (v2.0.0) (55). Maximum likelihood phylogeny was inferred with FastTree (v2.1.11) in the WAG+GAMMA model (60). The phylogenetic tree was then visualized and annotated on the iTOL (https://itol.embl.de/) (61).

### Function annotation

Open reading frames (ORFs) in the above genomes were predicted using Prokka (v1.14.6) (62) with default parameters. All ORFs were annotated using the Kyoto Encyclopedia of Genes and Genomes database with GhostKOALA (63) and against the Pfam (release 35.0) (64), NCBIfam (release 11.0) (65), and SUPERFAMILY (release 1.75) (66) HMM models using Interproscan (v5.62-94.0) (67). All genomes were also submitted to the METABOLIC v2.0 annotation pipeline for functional annotation (68). Detailed descriptions of the cell structure, motility, metabolic reconstruction, hydrogenase identification and classification, and ALE can be found in Text S1.

## Data Availability

All data generated or analyzed during this study are included in this published article and its supplementary files.

## SUPPLEMENTAL MATERIAL

Data Set S1 msystems.00999-24-s0001.xlsx Metagenomic analysis results.

Text S1 msystems.00999-24-s0002.pdf Supplemental methods, notes, and legends.

Fig. S1 msystems.00999-24-s0003.pdf Distribution of *Hydrogenedentota* around the globe.

Fig. S2 msystems.00999-24-s0004.pdf Relative abundance of *Hydrogenedentota* in different habits.

Fig. S3 msystems.00999-24-s0005.pdf Phylogenomic tree of *Hydrogenedentota*.

Fig. S4 msystems.00999-24-s0006.pdf Classification of [FeFe]-hydrogenases and [NiFe]-hydrogenases.

Fig. S5 msystems.00999-24-s0007.pdf Organization of 195 gene clusters coding for BfuABC homologs.

Fig. S6 msystems.00999-24-s0008.pdf Unrooted radiation tree of the subunits BfuA, BfuB, and BfuC.

Fig. S7 msystems.00999-24-s0009.pdf Schematic diagram of H-cluster motifs.

Fig. S8 msystems.00999-24-s0010.pdf Events of inferred duplications, intra-phylum transfers, losses and gene originations.

ASM does not own the copyrights to Supplemental Material that may be linked to, or accessed through, an article. The authors have granted ASM a non-exclusive, world-wide license to publish the Supplemental Material files. Please contact the corresponding author directly for reuse.
