# Supplementary material for: Diverse non-canonical electron bifurcating [FeFe]-hydrogenases of separate evolutionary origins in Hydrogenedentota
Source: mSystems. 2024 Aug 27;9(9):e00999-24. doi: 10.1128/msystems.00999-24 (PMC11406978; doi:10.1128/msystems.00999-24)
Supplement: Text S1 — Supplemental methods, notes, and legends. [file msystems.00999-24-s0002.pdf]

Supplementary Information for

Members of *Hydrogenedentota* have evolved diverse electron bifurcating [FeFe]-hydrogenases with non-canonical catalytic mechanism to enhance survival

Xiaowei Zheng[a] and Li Huang[a,b]

[a] State Key Laboratory of Microbial Resources, Institute of Microbiology, Chinese Academy of Sciences, Beijing 100101, P.R. China

[b] College of Life Sciences, University of Chinese Academy of Sciences, Beijing 100049, P. R. China

This file includes:

1. **Supplementary Methods 2**
   - 1.1 Cell structure and motility 2
   - 1.2 Metabolic reconstruction 2
   - 1.3 Identification and phylogenetic analysis of hydrogenases 4
   - 1.4 Amalgamated likelihood estimation (ALE). 6
2. **Supplementary Notes 6**
   - 2.1 Supplementary Note 1: Motility of *Hydrogenedentota* 6
   - 2.2 Supplementary Note 2: Metabolic potential of *Hydrogenedentota* 7
   - 2.3 Supplementary Note 3: [NiFe] hydrogenases in *Hydrogenedentota* 9
3. **Legends of Supplementary Data Set S1, Sheet 1 to 10 .... 12**
4. **Legends of Supplementary Figures S1 to S8 .... 12**
5. **Supplementary References 15**

1. Supplementary Methods

1.1 Cell structure and motility. The Raetz pathway for lipopolysaccharide biosynthesis was considered complete if the completeness of M00060 module (KDO2-lipid A biosynthesis, LpxL-LpxM typ) was over 60% (partial: 30 - 60%; absent: <30%). UDP-*N*-acetylmuramoyl-L-alanyl-D-glutamate: *meso*-2,6-diaminopimelate ligase (K01928) and UDP-*N*-acetylmuramoyl-L-alanyl-D-glutamate: L-lysine ligase (K05362) were used to infer the type of pentapeptide in disaccharide pentapeptide monomer, which determines the cell wall structure through the third amino acid residue, *meso*-diaminopimelic acid (*m*DAP) or L-lysine (1). Any of the chromosome and associated proteins (rodA: K05837, mreB: K03569, mreC: K03570, or mreD: K03571) was used to predict a cell shape. At least four of the eight types of flagellar assembly proteins (Type-III secretion, C ring, M, S, P and L rings, Rod and hook, H and T rings, Filament, Stator and Others), and two of the four type IV pilus assembly proteins PilABCD (K02650, K02652, K02653 and K02654), were used to postulate the existence of flagella and pilus, respectively (Data Set S1, Sheet 3). Chemotaxis was investigated by assessing the completeness of the two component system proteins (> 60%) and presence of methyl-accepting chemotaxis protein (MCP, K03406, Data Set S1, Sheet 3).

1.2 Metabolic reconstruction. Potential metabolic pathways were reconstructed based on KEGG annotations as described previously (2). Briefly, the Embden–Meyerhof–Parnas pathway (EMP, module M00001), the pentose phosphate pathway (PPP, modules M00006 and M00007), the Entner–Doudoroff pathway (ED, module M00008) and pyruvate oxidation (module M00307) were considered complete if the completeness of their corresponding KEGG modules were over 80% (partial: 50 - 80%; absent: <50%). The tricarboxylic acid (TCA) cycle was considered complete if 70% of the key enzymes represented by their KEGG accessions were present

(partial: 50 - 70%, absent: <50%, Data Set S1, Sheets 4 and 5). Glyoxylate cycle was considered complete if both isocitrate lyase (aceA: K01637) and malate synthase (aceB: K01638) were annotated in the genome. Gluconeogenesis pathway was considered complete if all of the three key enzymes were present (partial if anyone; absent if none): phosphoenol pyruvate carboxykinase (*pck*A, K01610 or K01596), fructose-1,6-bisphosphatase (*fbp*: K03841) and glucose phosphatase (agp: K01085 or G6PC: K01084). Both the starch/glycogen synthase (K00703, K20812 or K13679) and 1,4-alpha-glucan branching enzyme (K00700 or K16149), were used for inferring the capacity of synthesizing common energy-storage polysaccharides (starch or glycogen). And the capacity of synthesizing trehalose was considered present if any one of followed proteins was detected: alpha-D-glucosyltransferase (treS: K05343), maltooligosyltrehalose trehalohydrolase (treZ: K01236), trehalose synthase (treT: K13057), trehalose 6-phosphate synthase (otsA: K00697), trehalose 6-phosphate synthase/phosphatase (TPS:K16055), trehalose 6-phosphate synthase complex regulatory subunit (TSL1: K22337), sugar PTS system EIIA component (crr: K02777), trehalose PTS system EIIBC or EIIBCA component (treB, treP: K02819). The butyrate fermentation pathway was considered complete in a genome if the two subunits of the acetate CoA/acetoacetate CoA transferase (K01034 and K01035), the butyrate kinase (K00929) and the butyryl-CoA dehydrogenase (K00248) were present. The acetate metabolism pathway was considered complete if both the acetate/propionate kinase (K00925 or K00932) and the phosphate acetyltransferase (K00625, K13788 or K15024) were present or if the two subunits of the acetate-CoA ligase were present (K01905 and K22224). The ethanol fermentation pathway was considered complete if both the aldehyde dehydrogenase (K00128, K00129, K14085, K00149 or K00138) and the alcohol dehydrogenase (K00114, K13951, K13980, K13952, K13953, K13954, K00001, K00121 or K18857) were

present or if the multifunctional aldehyde-alcohol dehydrogenase (encoded by the adhE gene, K04072) that catalyzes the sequential reduction of acetyl-CoA to acetaldehyde and then to ethanol under fermentative conditions was present. Lactate fermentation was considered present if the lactate dehydrogenase was present (K00016) and malate fermentation if malate dehydrogenase was present (K00024).

Electron transport complex I (NADH-quinone oxidoreductase, M00144), complex II (Succinate dehydrogenase, M00149), complex IV (Cytochrome c oxidase: M00155; Cytochrome bd ubiquinol oxidase: M00153) and complex V (F-type ATPase: M00157; V/A-type ATPase: M00159) were considered complete if the completeness of their corresponding KEGG modules were over 60% (partial: 30 - 60%; absent: <30%). The *Rhodobacter* nitrogen fixation complex (RnfABCDEG, represented by the KEGG accessions K03617, K03616, K03615, K03614, K03613 and K03612) and sodium-pumping NADH: ubiquinone oxidoreductase (NqrABCDEF, represented by the KEGG accessions K00346, K00347, K00348, K00349, K00350 and K00351), were considered complete in a genome if at least 4 out of 6 subunits were found in operon along the genome, respectively. Nitrate reductase (NarGHI, K00370+K00371+K00374), nitrite oxidoreductase (NxrAB, K00370+K00371), nitrite reductase (NrfAH, K03385, K15876), and nitric oxide reductase (NorBC, K04561+K02305) were considered existed in a genome if any above subunit was annotated, respectively.

1.3 Identification and phylogenetic analysis of hydrogenases. A reference set of amino acid sequences corresponding to 3,265 hydrogenases was downloaded from the HydDB database (3). A local hydrogenase database was built using only 1,941 [NiFe]- and 1,220 [FeFe]-hydrogenase sequences (Data Set S1, Sheets 8 and 9), which contain conserved cysteine residues required to ligate H_2-binding metal centers, to reduce the risk of mis-annotation resulting from the presence

of various protein families containing hydrogenase homologs unable to metabolize H_2 (4). Predicted ORFs were first aligned against the local reference hydrogenase database using Diamond (v2.0.15.153; option: --id 30 --evalue 0.01 --query-cover 50 --subject-cover 50) (5). The amino acid sequences of putative hydrogenases identified in these searches were uploaded to the HydDB webserver (3) to functionally classify hydrogenases and remove non-hydrogenases, and the results were manually validated through screening of the metal-binding motifs reported previously (i.e., motifs L1 and L2 in [NiFe]-hydrogenases, and motifs P1, P2 and P3 in [FeFe]-hydrogenases) (6-8)). After further annotation by Interproscan (v5.62-94.0) (9), high-confidence hydrogenases containing the “*Ni-dep_hyd_lsu*” domain (for the large subunit of [NiFe]-hydrogenases) and “*Fe_hydrogenase_lsu_C*” domain (for the H-cluster of [FeFe]-hydrogenases) were selected for the analysis of their neighboring genetic organization using ‘gggenes’ package (v0.5.1) in R program (10). The structural model of hydrogenase subunits was constructed by homology modeling with SwissModel (11) and visualized using VMD (v1.9.2) (12). [NiFe]-hydrogenase maturation proteins (hypABCDEF) was considered present if at least four of the six enzymes detected from Interproscan results (partial: 2~4, absent: <2). [FeFe]-hydrogenase maturation proteins (hydEFG) was considered present if at least two of the four enzymes detected from Interproscan results (partial: 1~2, absent: <1).

For further classification, a total of 492 [FeFe]-hydrogenase and 82 [NiFe]-hydrogenase identified in the 179 genomes were merged with their top 5 ‘diamond blastp’ alignments from the retained reference set of 3,161 hydrogenases (Data Set S1, Sheets 8 and 9), and phylogenetic trees were built as follows. Hydrogenase sequences were first aligned with MAFFT (v7.505) (13) using default parameters. The resulting alignments were trimmed using TrimAl (v1.4.rev15; option: -automated1) (14) before a phylogenetic tree was constructed by IQ-TREE (v1.6.12) (15)

in the LG + R10 model with 1,000 ultrafast bootstraps replicates. In addition, ORFs encoding the three subunits (BfuA, BfuB and BfuC) of [FeFe]-hydrogenase were aligned individually against proteins downloaded from NCBI RefSeq genome database (ftp://ftp.ncbi.nlm.nih.gov/genomes/refseq/bacteria/, July. 2023) using Diamond (v2.0.15.153) with default parameters (5). Target proteins with the highest BLASTp scores at species level were selected, and the three subunits from the same genome were concatenated for phylogeny analysis as described above.

1.4 Amalgamated likelihood estimation (ALE). Orthogroups were predicted in 90 non-redundant species-level genomes (Data Set S1, Sheet 2), using OrthoFinder (v2.5.5) with default settings (16). The resulting gene family trees were probabilistically reconciled against above phylogenetic tree of 120 bacterial marker proteins, to infer the frequencies of duplications, intra-LGTs (gene transfers within sampled genomes or species tree), losses, and originations (gene transfers outside sampled genomes or species tree, or de novo gene formation), using ALEml_undated in ALE package (v1.0) (17). Genome incompleteness estimated by CheckM (v1.1.9) (18), was probabilistically accounted for within ALE. The resulting '.uml_rec' files of ALE output were parsed to infer the mechanism of gene content change throughout the whole phylum, using the Python script of 'branchwise_numbers_of_events.py' (19).

2. Supplementary Notes

2.1 Supplementary Note 1: Motility of *Hydrogenedentota*

All the members of *Hydrogenedentota* contain pilus assembly-encoding genes, and 72% genomes contain flagellar assembly-encoding genes (Fig. 2). Except for genomes from Clade 6,

chemotaxis is prevalent in *Hydrogenedentota*, as the possession of two component system proteins and methyl-accepting chemotaxis proteins were detected in at least 54 and 44 non-redundant strains, respectively, from the other six clades (Fig. 2).

2.2 Supplementary Note 2: Metabolic potential of *Hydrogenedentota*

The breakdown of glucose to pyruvate, can be achieved through EMP (Embden–Meyerhof–Parnas pathway, usually called glycolysis), and almost all *Hydrogenedentota* genomes in this study have a complete or near-complete EMP pathway. Specifically, genes in the EMP pathway (M00001 module) are annotated in 98 of the 100 non-redundant genomes, and at least 65 genomes are predicted to have a complete EMP pathway, showing glycolysis is a main process for breaking down glucose to pyruvate (Fig. 2; Data Set S1, Sheets 4 and 5). And those genomes lacking a complete EMP pathway may acquire pyruvate via the ED (Entner–Doudoroff) pathway as an alternative since a gene encoding the key enzyme of 2-dehydro-3-deoxyphosphogluconate aldolase (*eda*) was found in over 80% *Hydrogenedentota* genomes (Data Set S1, Sheet 4 and Fig. 2). And over 82% of the *Hydrogenedentota* genomes may have the pentose phosphate pathway (PPP), which provides an alternative to glycolysis for glucose oxidation and essential substrates (*e.g.*, NADPH and pentose phosphate) for the synthesis of nucleotides, amino acids, cofactors and vitamins. The pyruvate oxidation (M00307 module) pathway, a connector that links glycolysis to TCA by providing the fuel of acetyl Coenzyme A (acetyl CoA), was observed in over 98% of the genomes (Fig. 2). Coupled with these metabolic features, the possession of TCA cycle in at least 89% of the genomes further indicates that *Hydrogenedentota* most likely live a heterotrophic lifestyle.

29 non-redundant genomes in Clade 1 are predicted to have the acetate fermentation ability, which has seldom been detected in other clades. Ethanol fermentation appears to be more widely

distributed in *Hydrogenedentota* than acetate production since 47 non-redundant genomes from all the seven clades might produce ethanol as byproduct in anaerobic conditions (Fig. 2). The continuation of glycolysis depends upon the availability of the oxidized form of the electron carrier, NAD^+. Thus, NADH must be continuously oxidized back into NAD^+ to keep glycolysis going under anaerobic conditions. In addition to the above-mentioned possible fermentation processes, an alternate solution might be realized by L-lactate dehydrogenase (LDH, ldh; [EC:1.1.1.27]), catalyzing the reduction of pyruvate to lactate and regenerate NAD^+. However, the LDH encoding gene has only been detected in 14% of the non-redundant genomes mainly from Clade 1 (Fig. 2). Alternatively, pyruvate formed via glycolysis could be fermented to lactate by one of the two lactate dehydrogenases (K00016 or K03778), producing NADH, and the lactate excreted from the cell. Notably, the balance of NAD^+ and NADH might also be temporarily adjusted by malate dehydrogenase (MDH, l-malate:NAD oxidoreductase, EC 1.1.1.37) for its ability to catalyze the NAD^+/NADH-dependent interconversion between malate and oxaloacetate, and it may function in almost 80% of the genomes of *Hydrogenedentota* (Data Set S1, Sheets 4 and 5). Based on these data, we speculate that members of *Hydrogenedentota* are heterotroph and able to produce ATP via substrate level phosphorylation under anaerobic conditions.

In addition, the glyoxylate cycle, a special variant of the tricarboxylic cycle (TCA) that allows utilization of two carbons compounds in the absence of glucose, are complete in about 26% of the non-redundant genomes (Fig. 2). Instead of converting isocitrate to α-ketoglutarate in TCA cycle, the glyoxylate cycle enzyme isocitrate lyase (EC 4.1.3.1) catalyzes the conversion of isocitrate (C6) into glyoxylate (C2) and succinate (C4). Subsequently, malate synthase (EC 2.3.3.9) catalyzes the condensation of glyoxylate with acetyl-CoA (C2) to produce malate (C4)

and a free CoA molecule. Malate can be further oxidized into oxaloacetate, an important precursor for gluconeogenic biosynthesis of glucose and other sugars. However, no genomes containing all of the three key enzymes of gluconeogenesis:phosphoenol pyruvate carboxykinase (*pck*A, K01596, K01610), fructose-1,6-bisphosphatase (*fbp*, K02446) and glucokinase (*glk*, K12407, K25026). And the proportion of genomes containing at least one of these three rate-limiting enzymes is about 82%, 1% and 96%, respectively. Obviously, the lack of fructose-1,6-bisphosphatase in most members of *Hydrogenedentota* blocks the pathway of gluconeogenesis. Moreover, two genes encoding starch synthase [EC:2.4.1.21] and 1,4-alpha-glucan branching enzyme, which are responsibly for starch or glycogen synthesis, were detected together only in 12 non-redundant genomes. And trehalose, known as a protective agent that help cells adapt to cold and high-pressure habitats, is only synthesized by fewer than 10% of the members of *Hydrogenedentota*. Therefore, the process of gluconeogenesis storing sufficient energy in glucose, followed by the synthesis of more complex compounds (*e.g.*, glycogen, starch and trehalose), may not occur in *Hydrogenedentota*.

2.3 Supplementary Note 3: [NiFe] hydrogenases in *Hydrogenedentota*

We identified six types of [NiFe]-hydrogenases (groups 1a, 1c, 3b, 3c, 3d and 4g) in *Hydrogenedentota* (Data Set S1, Sheet 12). Groups 1a [NiFe]-hydrogenases were only annotated in 6 genomes, which belong to Clade V (5) and Clade VI (1) (Data Set S1, Sheet 12, and Fig. 2), respectively. Similarly, groups 1c [NiFe]-hydrogenases were also only annotated in 6 genomes, all of which belong to Clade VI (Data Set S1, Sheet 12, and Fig. 2). These observations show that both groups 1a and 1c [NiFe]-hydrogenases are not prevalent in *Hydrogenedentota*. Unlike the genomes from cultured isolates, metagenome-assembled genomes (MAGs) are usually composed of contigs or scaffolds with varying lengths, some of which are even only ~2kb, thus

limiting our ability to speculate on which physiological processes both groups 1a and 1c [NiFe]-hydrogenases are involved in in *Hydrogenedentota* by inspecting their neighborhood coding-genes (Data Set S1, Sheet 12 and Fig. 2).

Group 3b [NiFe]-hydrogenases were annotated in 15 genomes, which belong to Clade I (8), Clade II (1), Clade IV (5) and Clade VII (1) (Data Set S1, Sheet 12 and Fig. 2). Flanking the genes encoding group 3b [NiFe]-hydrogenase in one *Hydrogenedentota* genome (GCA_016182645.1) is a gene encoding a pyruvate-ferredoxin/flavodoxin oxidoreductase (K03737, por/nifJ), which generates reduced ferredoxin during the conversion of pyruvate to acetyl-coA. The resulting reduced ferredoxin might be electron donor used by group 3b [NiFe]-hydrogenase to produce H_2. Two genes encoding a NAD(P)-binding subunit and hydrogenase small subunit, respectively, are adjacently located (Data Set S1, Sheet 12 and Fig. 2), suggesting that NAD(P) may be used by group 3b [NiFe]-hydrogenases as an electron acceptor. It appears that this type of hydrogenases may play an important role in conserving energy in the form of NAD(P)H for anabolism.

Like group 1a and group 1c [NiFe]-hydrogenases, group 3c [NiFe]-hydrogenases are not widely distributed in *Hydrogenedentota*, as only 4 genomes, which belong to Clade I (1), Clade III (1) and Clade VII (2), are detected having this type of hydrogenase (Data Set S1, Sheet 12 and Fig. 2). In contrast, group 3d [NiFe]-hydrogenases (HoxEFUYH) were annotated in 44 genomes. A half of them belong to Clade I (22), and the remainder to Clade III (2), Clade IV (4), Clade V (4) and Clade VII (12) (Data Set S1, Sheet 12 and Fig. 2). Group 3c [NiFe] methyl viologen-reducing (Mvh) hydrogenases are believed to be flavin-based electron bifurcating (FBEB) enzymes, which are different from group 3d [NiFe]-hydrogenases in *Hydrogenedentota*, whose electron bifurcation pathway involves a combination of FMN and adjacent iron sulfur

clusters (“FMN-FeS”) rather than a single FAD (20, 21). Group 3d [NiFe]-hydrogenases are generally believed to use NAD(P)H to reduce H^+ and accept electrons from ferredoxin during pyruvate fermentation (22). However, the lack of two FeS clusters in the hoxF/HydB subunit (a homolog of diaphorase (23) or BfuB subunit in group A3 [FeFe]-hydrogenase (20, 21)) suggests that group 3d [NiFe]-hydrogenase in *Hydrogenedentota* are unable to accept electrons from ferredoxin (Fig. S4f). Thus, group 3d [NiFe]-hydrogenases are unable to oxidize ferredoxin in *Hydrogenedentota*. Interestingly, the domain containing FeS clusters (like “B3B4” FeS clusters in BfuB) is also replaced by a dihydroprymidine dehydrogenase domain (DPD_II) with an extra FAD/NAD(P)-binding domain located at the C-terminus of hoxF subunit in three Clade I genomes (GCA_007134415.1, GCA_007126205.1 and GCA_007118235.1) (Fig. S4f). Whether these three hydrogenases are electron bifurcating enzymes awaits further verification as discussed in main text.

Only 6 genomes, which belong to Clade I (3), Clade IV (1) and Clade V (2), are found to have group 4g [NiFe]-hydrogenases (Data Set S1, Sheet 12 and Fig. 2). These hydrogenases, known as so-called energy-converting hydrogenases (Ech), couple the oxidation of ferredoxin with the formation of hydrogen, conserving energy in the form of an ion gradient through a Na^+/H^+ antiporter (Mrp) complex. This membrane-bound hydrogenase (Mbh) represents a simple respiratory system, further highlighting the diversity of energy conservation strategies in *Hydrogenedentota*.

3. Legends of Supplementary Data Set S1, Sheet 1 to 10

Data Set S1: Sheet 1. Relative abundance (%) of *Hydrogenedentota* in EMP samples based on 16S rRNA reads. **Sheet 2.** Detailed information of the 179 *Hydrogenedentota* genomes and MAGs. **Sheet 3.** Cell structure and chemotaxis of the 179 *Hydrogenedentota* genomes based on KEGG orthology (KO). **Sheet 4.** Percentage of genomes containing the corresponding KEGG orthology (KO). **Sheet 5.** KEGG pathways and related types of metabolism in each of the 179 *Hydrogenedentota* genomes. **Sheet 6.** Potential H-cluster ([FeFe]-hydrogenase active site) binding motifs of P1, P2 and P3 in *Hydrogenedentota* genomes. **Sheet 7.** 2,144 reference genomes containing BfuABC homologs. **Sheet 8.** 1,220 [FeFe]-hydrogenases from the HydDB database. **Sheet 9.** 1,941 [NiFe]-hydrogenases from the HydDB database. **Sheet 10.** Inferred duplications, intra-phylum transfers, losses and gene originations events based on 90 non-redundant species-level genomes of *Hydrogenedentota*. **Sheet 11.** Annotations of gene clusters containing group C1 [FeFe]-hydrogenase coding genes which are not located together with group A3 [FeFe]-hydrogenase coding genes (upstream: 1 to 10, downstream: -1 to -10). **Sheet 12.** Annotations of gene clusters containing [NiFe]-hydrogenase coding gene (upstream: 1 to 10, downstream: -1 to -10).

4. Legends of Supplementary Figures S1 to S8

Fig. S1. Distribution of *Hydrogenedentota* around the globe based on 16S rRNA high-throughput sequencing. Sites where *Hydrogenedentota* was detected (red) or not detected (blue) are indicated.

Fig. S2. Relative abundance (%) of *Hydrogenedentota* in different habits based on 16S rRNA genes.

Fig. S3. Phylogenomic tree of *Hydrogenedentota* inferred from 120 concatenated marker proteins. The tree was rooted with the genome of *Gimesia maris* DSM 8797 (GenBank assembly no. GCF_008298035.1). 100 non-redundant genomes are indicated by red stars. *Hydrogenedentota* clusters at seven family-level clades (Clade 1 to V7) are marked by colors according to Fig. 2. The bootstraps are labelled with black dots in the middle of branches when ≥ 90%. The nodes where the red triangle and square are located represent two sub-clades 6-1 and 6-2, which belong to Clade6.

Fig. S4. Classification of [FeFe]-hydrogenases and [NiFe]-hydrogenases. a: Phylogenetic analysis of 492 [FeFe]-hydrogenases with a local reference set of hydrogenases (Red squares). The bootstraps are labelled with black dots in the middle of branches when ≥ 90%. **b:** Ribbon diagram showing the predicted structures of five sub-types of BfuA subunits (Group A3 [FeFe]-hydrogenases from genomes of GCA_007118765.1). **c:** Phylogenetic analysis of 210 group C [FeFe]-hydrogenases. The bootstraps are labelled with black dots in the middle of branches when ≥ 90%. Circle 1 illustrates the length and domain characteristics of 210 group C [FeFe]-hydrogenases. In circle 2, the number in red dot (downstream) and blue square (upstream) represent the relative positions of genes encoding group C [FeFe]-hydrogenases to A3 [FeFe]-hydrogenases. Green pentagram represents the proximity of genes encoding group C [FeFe]-hydrogenases and [NiFe]-hydrogenase. Circle 3 shows the length of the contigs where coding genes of these hydrogenases are located. **d:** Ribbon diagram showing the predicted structures of group C1 [FeFe]-hydrogenases and group C3 [FeFe]-hydrogenases (sub-type IV and V BfuT subunits) in *Hydrogenedentota* (above) and

target hydrogenases with best hits (below), and their sequence identities are located at bottom. "HSP90", represents a histidine kinase/HSP90-like ATPase (*HATPase_C*) domain, details of the legends are shown in Fig. 3. **e:** Phylogenetic analysis of 82 [NiFe]-hydrogenases with a local reference set of hydrogenases (Red squares). The bootstraps are labelled with black dots in the middle of branches when ≥ 90%. **f:** Phylogenetic analysis of group 3c and 3d [NiFe]-hydrogenases. The bootstraps are labelled with black dots in the middle of branches when ≥ 90%. The length and domain characteristics of electron bifurcating homologs of group 3c (4) and 3d (44) [NiFe]-hydrogenases are illustrated in middle. The bar chart in right side shows the length of the contigs where coding genes of these hydrogenases are located.

Fig. S5. Organization of 195 gene clusters (five sub-types) coding for BfuABC homologs in *Hydrogenedentota*. a: 45 sub-type I gene clusters; b: 30 sub-type II gene clusters; c: 47 sub-type III gene clusters; d: 25 sub-type IV gene clusters; e: 48 sub-type V gene clusters. The five sub-types of gene clusters are marked as in Fig. 3.

Fig. S6. Unrooted radiation tree of the subunits BfuA (left), BfuB (middle) and BfuC (right) from 195 nearly complete Group A3 [FeFe]-hydrogenases. Numbers in parentheses represent the number of the subunit in corresponding clades or types.

Fig. S7. Schematic diagram indicating the extent of conservation in residues comprising the H-cluster motifs (P1, P2, and P3) of five sub-types of BfuA. Residues with higher bit scores (*i.e.*, larger font sizes) suggest a greater degree of conservation at a given aligned position. The features of a given amino acid are show with colors.

Fig. S8. The boxplots represent events of inferred duplications, intra-phylum transfers, losses and gene originations during the evolution of *Hydrogenedentota*.

5. Supplementary References

1. Idrees M, Mohammad AR, Karodia N, Rahman A. 2020. Multimodal Role of Amino Acids in Microbial Control and Drug Development. Antibiotics 9:330. https://doi.org/10.3390/antibiotics9060330.
2. Méheust R, Castelle CJ, Matheus Carnevali PB, Farag IF, He C, Chen LX, Amano Y, Hug LA, Banfield JF. 2020. Groundwater Elusimicrobia are metabolically diverse compared to gut microbiome Elusimicrobia and some have a novel nitrogenase paralog. ISME J 14:2907-2922. https://doi.org/10.1038/s41396-020-0716-1.
3. Søndergaard D, Pedersen CN, Greening C. 2016. HydDB: A web tool for hydrogenase classification and analysis. Sci Rep 6:34212. https://doi.org/10.1038/srep34212.
4. Yu H, Schut GJ, Haja DK, Adams MWW, Li H. 2021. Evolution of complex I-like respiratory complexes. J Biol Chem 296:100740. https://doi.org/10.1016/j.jbc.2021.100740.
5. Buchfink B, Xie C, Huson DH. 2015. Fast and sensitive protein alignment using DIAMOND. Nat Methods 12:59-60. https://doi.org/10.1038/nmeth.3176.
6. Poudel S, Tokmina-Lukaszewska M, Colman DR, Refai M, Schut GJ, King PW, Maness PC, Adams MW, Peters JW, Bothner B, Boyd ES. 2016. Unification of [FeFe]-hydrogenases into three structural and functional groups. Biochim Biophys Acta 1860:1910-1921. https://doi.org/10.1016/j.bbagen.2016.05.034.
7. Vignais PM, Billoud B. 2007. Occurrence, classification, and biological function of hydrogenases: an overview. Chem Rev 107:4206-4272. https://doi.org/10.1021/cr050196r.

8. Vignais PM, Billoud B, Meyer J. 2001. Classification and phylogeny of hydrogenases. FEMS Microbiol Rev 25:455-501. https://doi.org/10.1111/j.1574-6976.2001.tb00587.x.

9. Jones P, Binns D, Chang HY, Fraser M, Li W, McAnulla C, McWilliam H, Maslen J, Mitchell A, Nuka G, Pesseat S, Quinn AF, Sangrador-Vegas A, Scheremetjew M, Yong SY, Lopez R, Hunter S. 2014. InterProScan 5: genome-scale protein function classification. Bioinformatics 30:1236-1240. https://doi.org/10.1093/bioinformatics/btu031.

10. Wilkins D, Kurtz Z. 2023. gggenes: Draw Gene Arrow Maps in 'ggplot2'. https://wilkox.org/gggenes/. Accessed

11. Waterhouse A, Bertoni M, Bienert S, Studer G, Tauriello G, Gumienny R, Heer FT, de Beer TAP, Rempfer C, Bordoli L, Lepore R, Schwede T. 2018. SWISS-MODEL: homology modelling of protein structures and complexes. Nucleic Acids Res 46:W296-W303. https://doi.org/10.1093/nar/gky427.

12. Humphrey W, Dalke A, Schulten K. 1996. VMD: visual molecular dynamics. J Mol Graph 14:33-38. https://doi.org/10.1016/0263-7855(96)00018-5.

13. Katoh K, Standley DM. 2013. MAFFT multiple sequence alignment software version 7: improvements in performance and usability. Mol Biol Evol 30:772-780. https://doi.org/10.1093/molbev/mst010.

14. Capella-Gutiérrez S, Silla-Martínez JM, Gabaldón T. 2009. trimAl: a tool for automated alignment trimming in large-scale phylogenetic analyses. Bioinformatics 25:1972-1973. https://doi.org/10.1093/bioinformatics/btp348.

15. Nguyen LT, Schmidt HA, von Haeseler A, Minh BQ. 2015. IQ-TREE: a fast and effective stochastic algorithm for estimating maximum-likelihood phylogenies. Mol Biol Evol 32:268-74. https://doi.org/10.1093/molbev/msu300.

16. Emms DM, Kelly S. 2019. OrthoFinder: phylogenetic orthology inference for comparative genomics. Genome Biol 20:238. https://doi.org/10.1186/s13059-019-1832-y.

17. Szöllõsi GJ, Rosikiewicz W, Boussau B, Tannier E, Daubin V. 2013. Efficient exploration of the space of reconciled gene trees. Syst Biol 62:901-912. https://doi.org/10.1093/sysbio/syt054.

18. Parks DH, Imelfort M, Skennerton CT, Hugenholtz P, Tyson GW. 2015. CheckM: assessing the quality of microbial genomes recovered from isolates, single cells, and metagenomes. Genome Res 25:1043-1055. https://doi.org/10.1101/gr.186072.114.

19. Sheridan PO, Raguideau S, Quince C, Holden J, Zhang L, Williams TA, Gubry-Rangin C. 2020. Gene duplication drives genome expansion in a major lineage of Thaumarchaeota. Nat Commun 11:5494. https://doi.org/10.1038/s41467-020-19132-x.

20. Schut GJ, Haja DK, Feng X, Poole FL, Li H, Adams MWW. 2022. An Abundant and Diverse New Family of Electron Bifurcating Enzymes With a Non-canonical Catalytic Mechanism. Front Microbiol 13:946711. https://doi.org/10.3389/fmicb.2022.946711.

21. Feng X, Schut GJ, Haja DK, Adams MWW, Li HL. 2022. Structure and electron transfer pathways of an electron-bifurcating NiFe-hydrogenase. Sci Adv 8:eabm7546. https://doi.org/10.1126/sciadv.abm7546.

22. Gutekunst K, Chen X, Schreiber K, Kaspar U, Makam S, Appel J. 2014. The bidirectional NiFe-hydrogenase in Synechocystis sp. PCC 6803 is reduced by flavodoxin and ferredoxin and is essential under mixotrophic, nitrate-limiting conditions. J Biol Chem 289:1930-1937. https://doi.org/10.1074/jbc.M113.526376.

23. Di Leonardo PF, Antonicelli G, Agostino V, Re A. 2022. Genome-Scale Mining of Acetogens of the Genus Clostridium Unveils Distinctive Traits in [FeFe]- and [NiFe]-

Hydrogenase Content and Maturation. Microbiol Spectr 10:e0101922. https://doi.org/10.1128/spectrum.01019-22.
