# Supplementary material for: Diverse non-canonical electron bifurcating [FeFe]-hydrogenases of separate evolutionary origins in Hydrogenedentota
Source: mSystems. 2024 Aug 27;9(9):e00999-24. doi: 10.1128/msystems.00999-24 (PMC11406978; doi:10.1128/msystems.00999-24)

Figure S1. Distribution of *Hydrogenedentota* around the globe based on 16S rRNA high-throughput sequencing

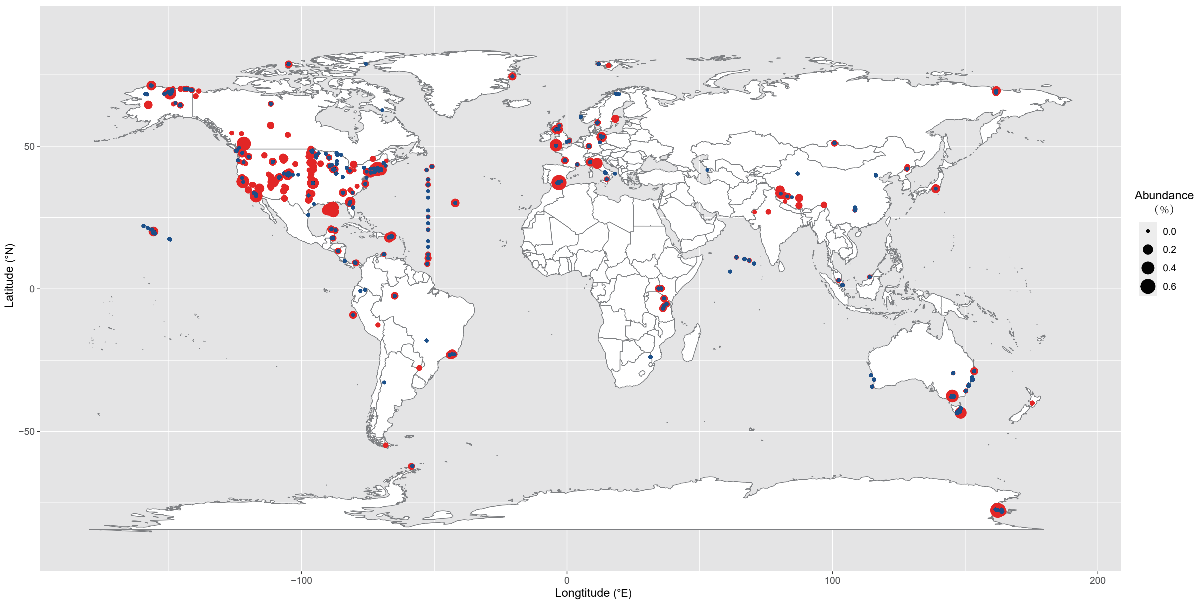

Supplement: Fig. S1 — Distribution of Hydrogenedentota around the globe. [file msystems.00999-24-s0003.pdf]
