# Supplementary figures and images for: Diverse non-canonical electron bifurcating [FeFe]-hydrogenases of separate evolutionary origins in Hydrogenedentota
Source: mSystems. 2024 Aug 27;9(9):e00999-24. doi: 10.1128/msystems.00999-24 (PMC11406978; doi:10.1128/msystems.00999-24)

Figure S2. Relative abundance (%) of *Hydrogenedentota* in different habits based on 16S rRNA genes

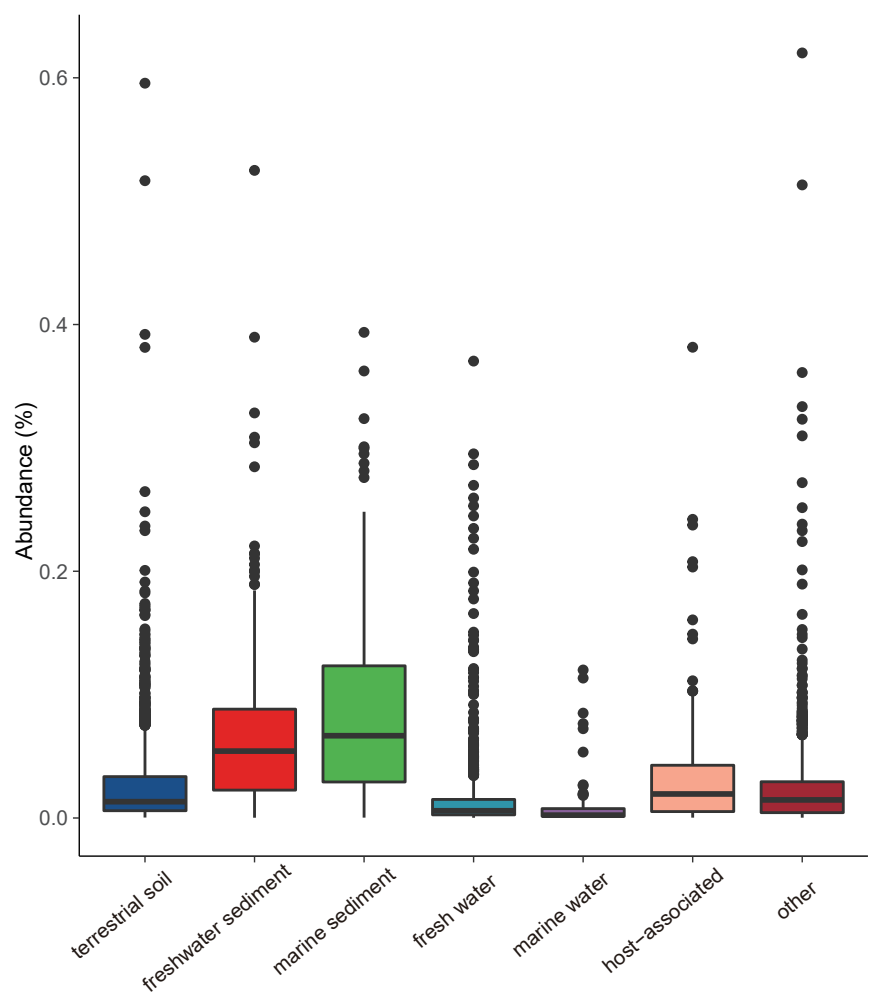

Supplement: Fig. S2 — Relative abundance of Hydrogenedentota in different habits. [file msystems.00999-24-s0004.pdf]

**Figure S3. Phylogenomic tree of *Hydrogenedentota* inferred from 120 concatenated marker proteins**

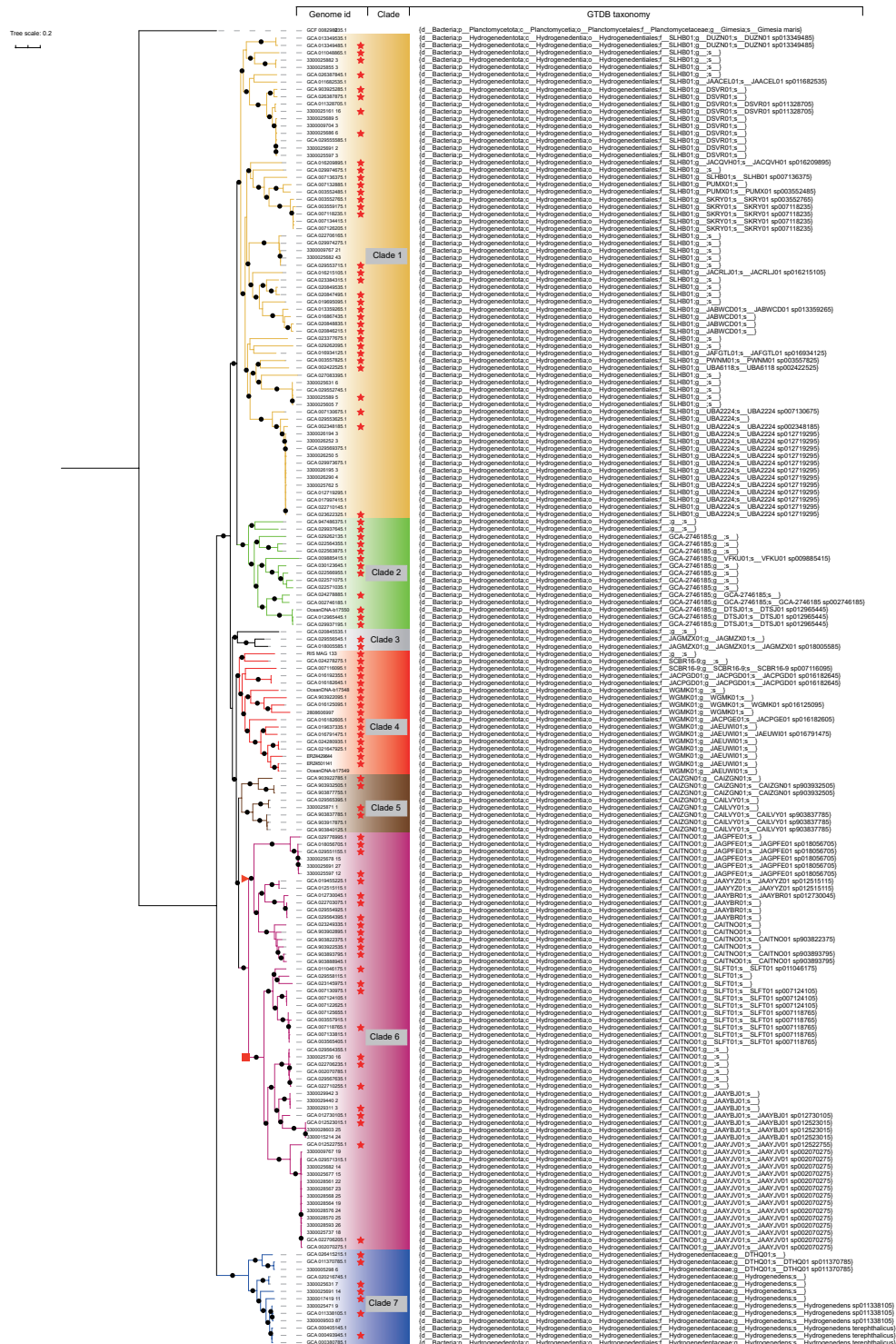

Supplement: Fig. S3 — Phylogenomic tree of Hydrogenedentota. [file msystems.00999-24-s0005.pdf]

**Figure S4. Classification of [FeFe]-hydrogenases and [NiFe]-hydrogenases**

**a**

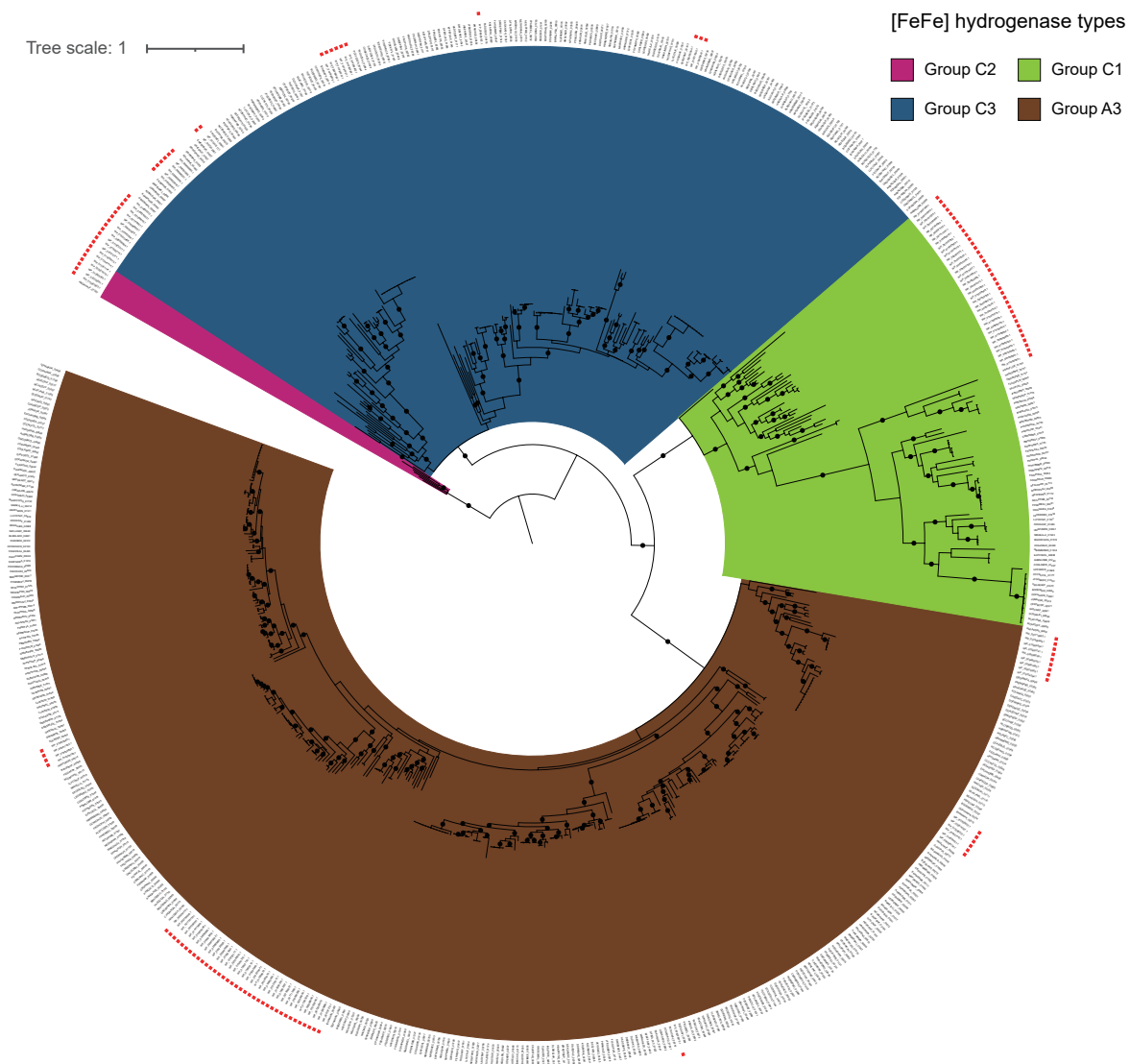

**b**

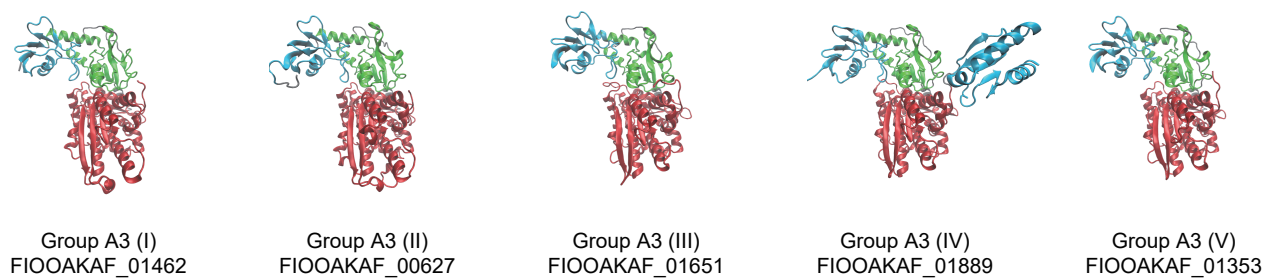

**c**

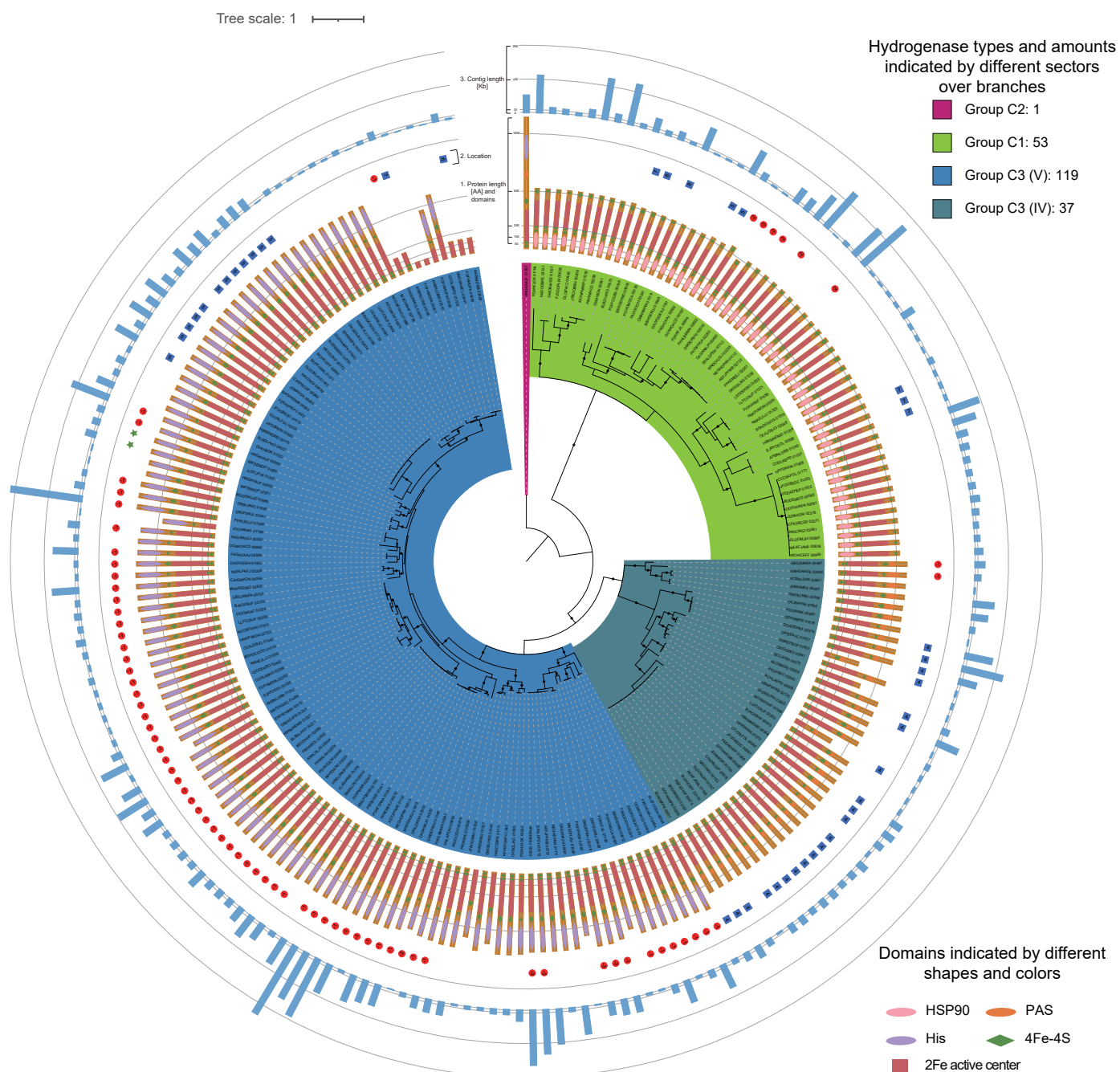

**d**

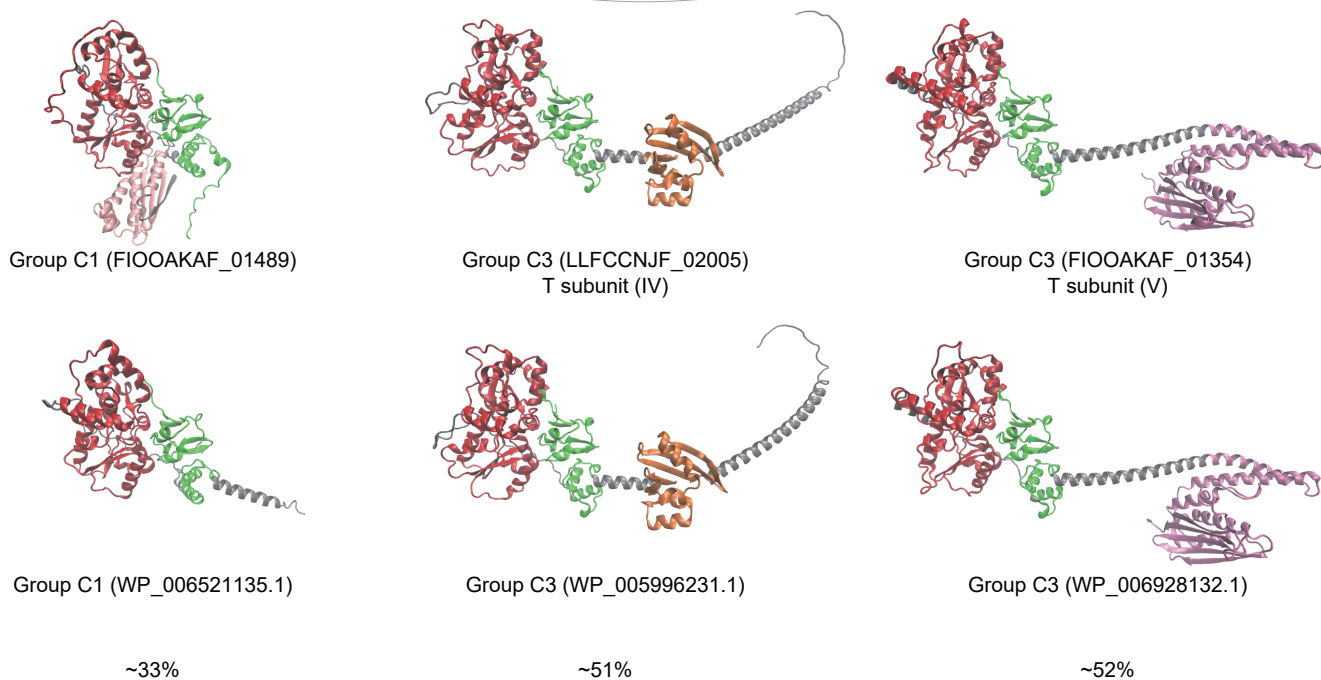

**e**

Tree scale: 1 

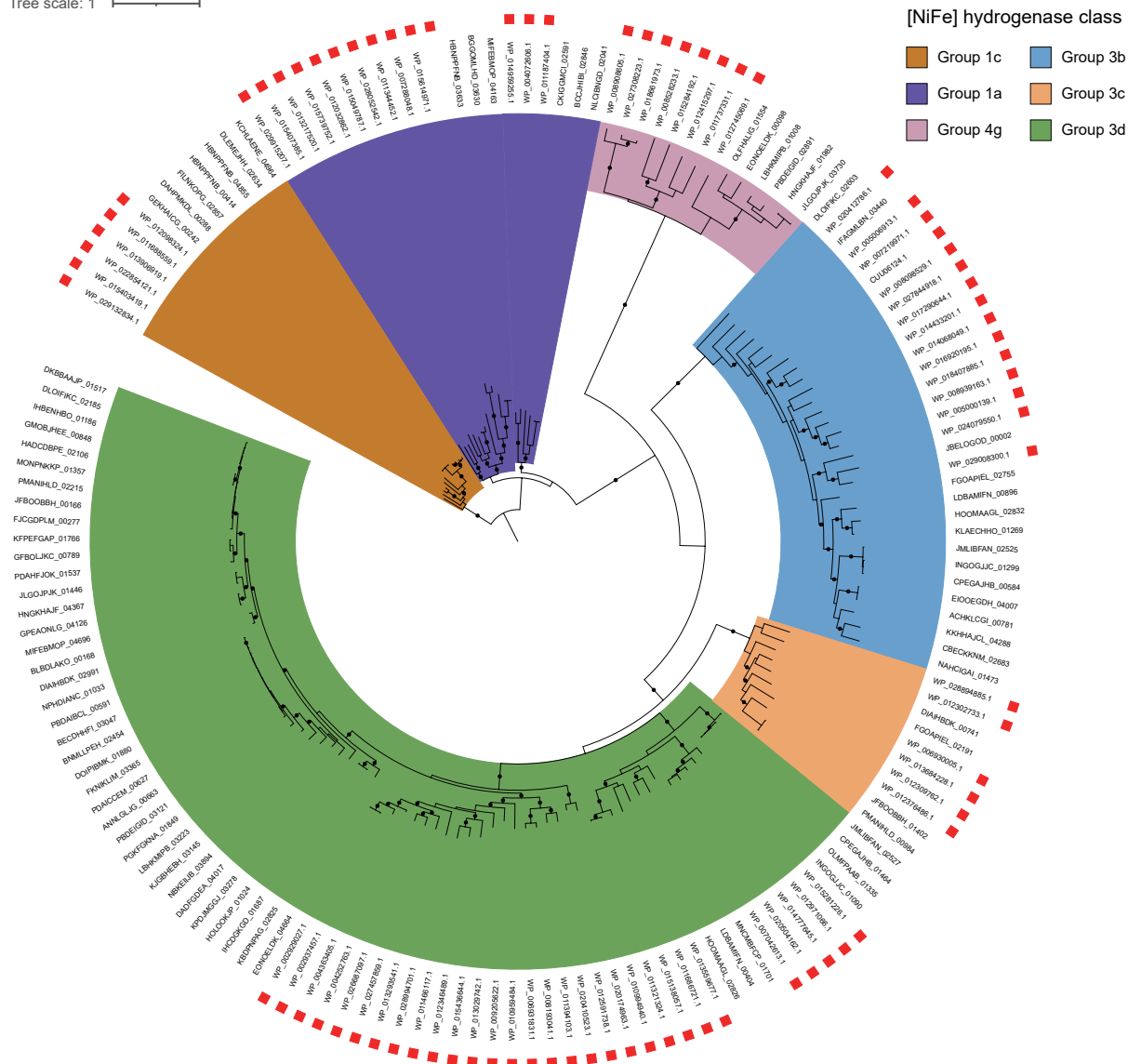

f

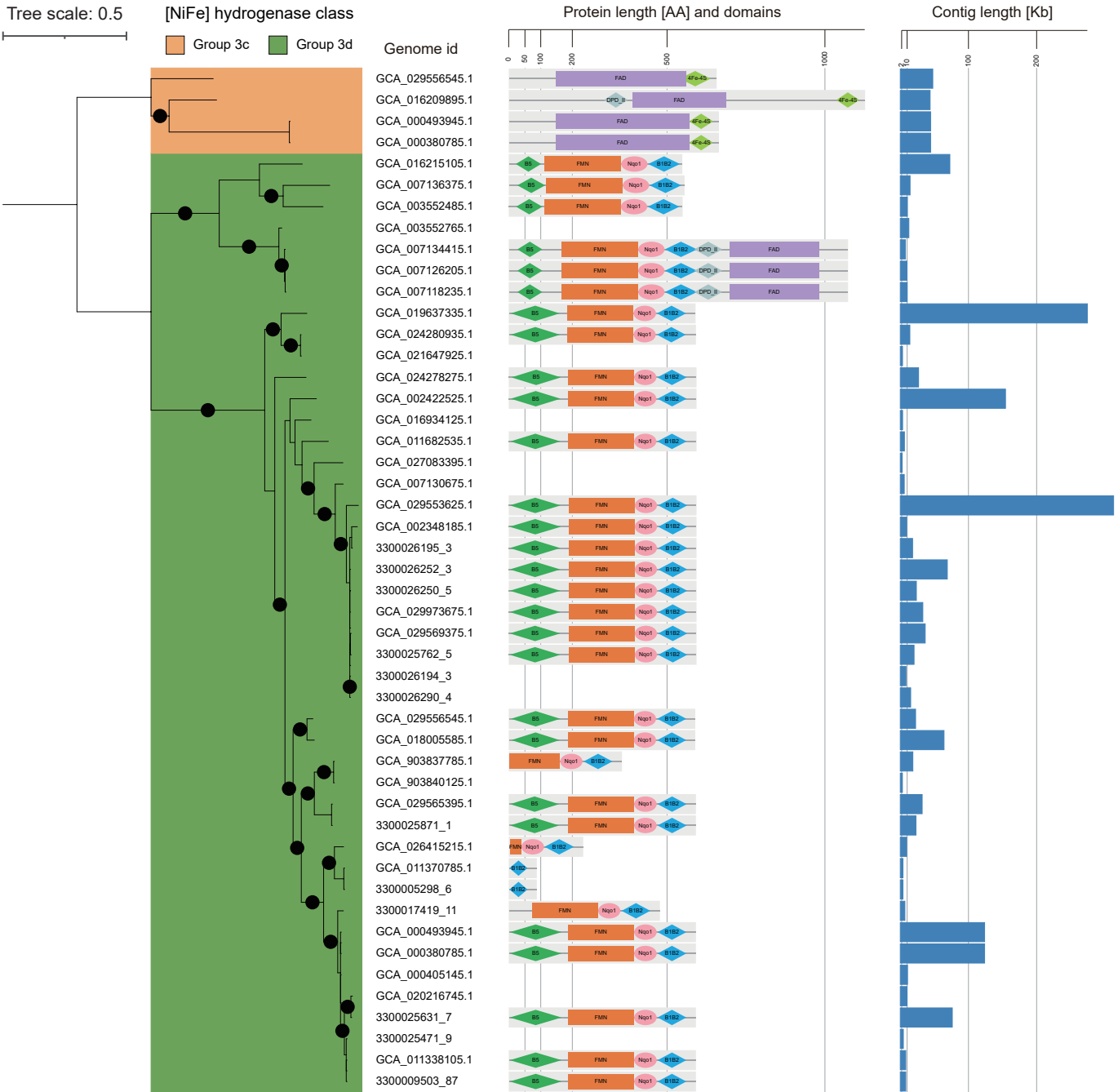

Supplement: Fig. S4 — Classification of [FeFe]-hydrogenases and [NiFe]-hydrogenases. [file msystems.00999-24-s0006.pdf]
