# Supplementary material for: Diverse non-canonical electron bifurcating [FeFe]-hydrogenases of separate evolutionary origins in Hydrogenedentota
Source: mSystems. 2024 Aug 27;9(9):e00999-24. doi: 10.1128/msystems.00999-24 (PMC11406978; doi:10.1128/msystems.00999-24)
Supplement: Fig. S5 — Organization of 195 gene clusters coding for BfuABC homologs. [file msystems.00999-24-s0007.pdf]

# Figure S5 Organization of 195 gene clusters (five sub-types) coding for BfuABC homologs in *Hydrogenedentota*

**a**

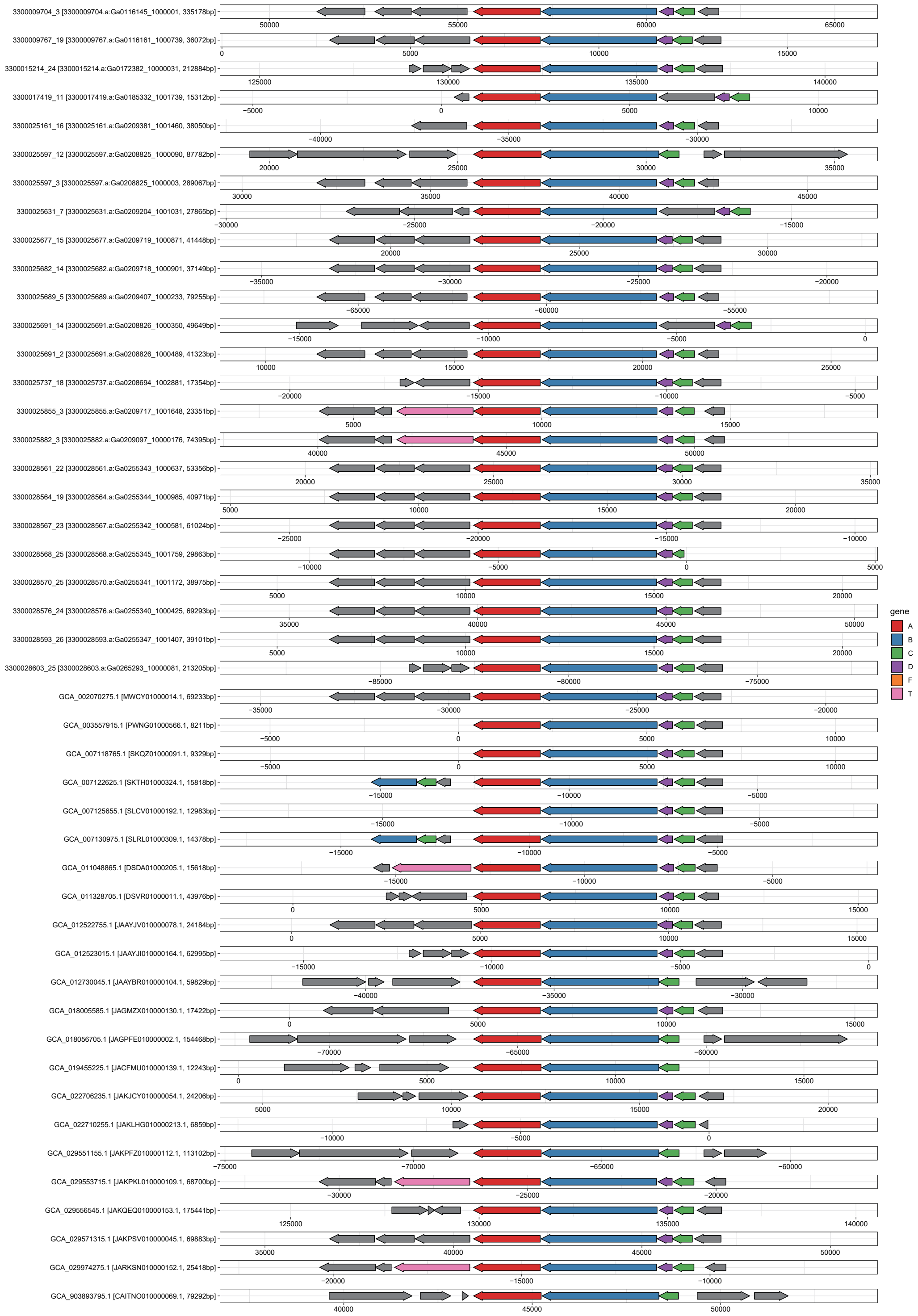

**b**

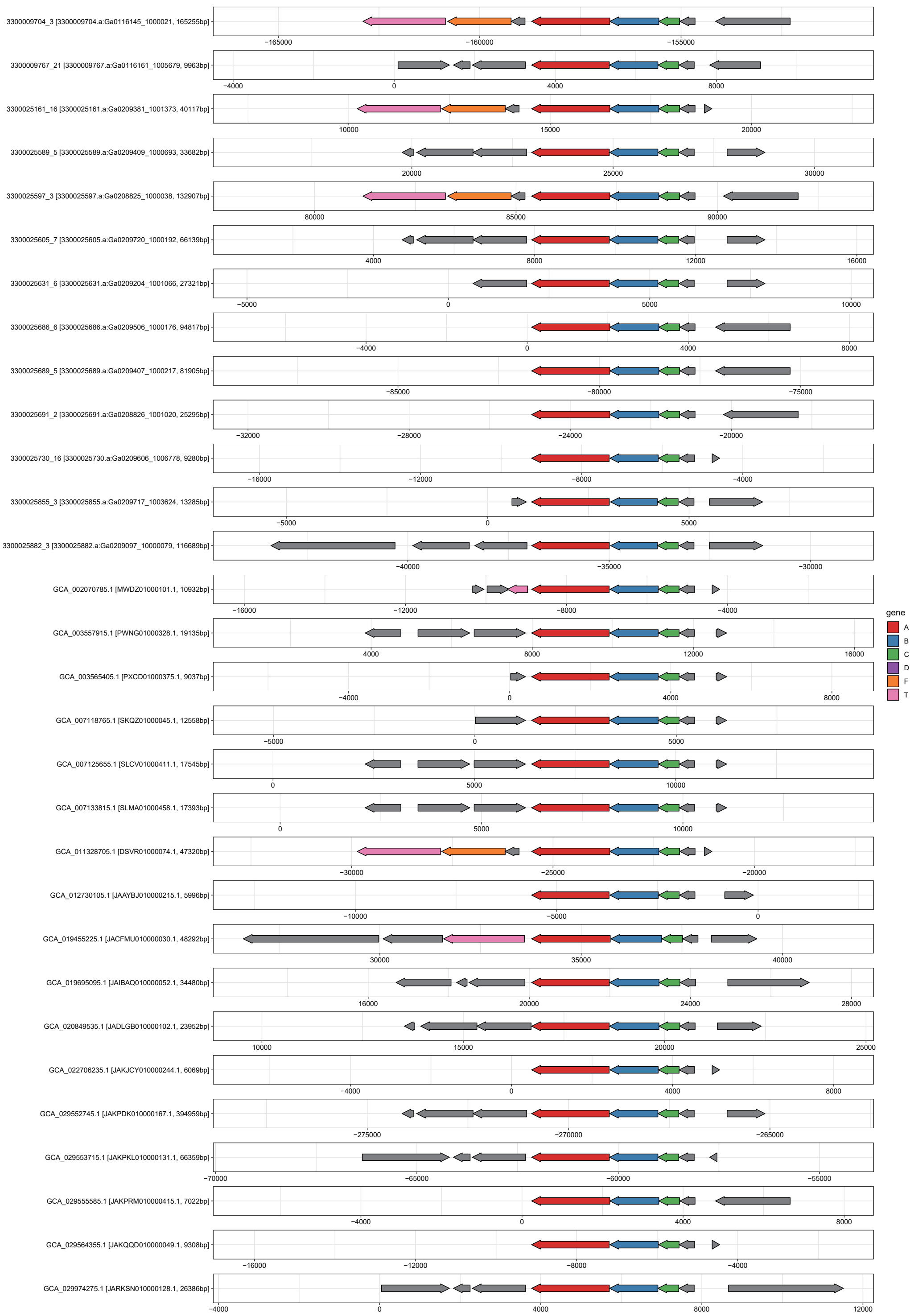
3300009704_3 [3300009704.a:Ga0116145_1000021, 165255bp]
3300009767_21 [3300009767.a:Ga0116161_1005679, 9963bp]
3300025161_16 [3300025161.a:Ga0209381_1001373, 40117bp]
3300025589_5 [3300025589.a:Ga0209409_1000693, 33682bp]
3300025597_3 [3300025597.a:Ga0208825_1000038, 132907bp]
3300025605_7 [3300025605.a:Ga0209720_1000192, 66139bp]
3300025631_6 [3300025631.a:Ga0209204_1001066, 27321bp]
3300025686_6 [3300025686.a:Ga0209506_1000176, 94817bp]
3300025689_5 [3300025689.a:Ga0209407_1000217, 81905bp]
3300025691_2 [3300025691.a:Ga0208826_1001020, 25295bp]
3300025730_16 [3300025730.a:Ga0209606_1006778, 9280bp]
3300025855_3 [3300025855.a:Ga0209717_1003624, 13285bp]
3300025882_3 [3300025882.a:Ga0209097_10000079, 116689bp]
GCA_002070785.1 [MWDZ01000101.1, 10932bp]
GCA_003557915.1 [PWNG01000328.1, 19135bp]
GCA_003565405.1 [PXCD01000375.1, 9037bp]
GCA_007118765.1 [SKQZ01000045.1, 12558bp]
GCA_007125655.1 [SLCV01000411.1, 17545bp]
GCA_007133815.1 [SLMA01000458.1, 17393bp]
GCA_011328705.1 [DSVR01000074.1, 47320bp]
GCA_012730105.1 [JAAYBJ010000215.1, 5996bp]
GCA_019455225.1 [JACFMU010000030.1, 48292bp]
GCA_019695095.1 [JAIBAQ010000052.1, 34480bp]
GCA_020849535.1 [JADLGB010000102.1, 23952bp]
GCA_022706235.1 [JAKJCY010000244.1, 6069bp]
GCA_029552745.1 [JAKPDK010000167.1, 394959bp]
GCA_029553715.1 [JAKPKL010000131.1, 66359bp]
GCA_029555585.1 [JAKPRM010000415.1, 7022bp]
GCA_029564355.1 [JAKQQD010000049.1, 9308bp]
GCA_029974275.1 [JARKSN010000128.1, 26386bp]
gene
A
B
C
D
F
T

c

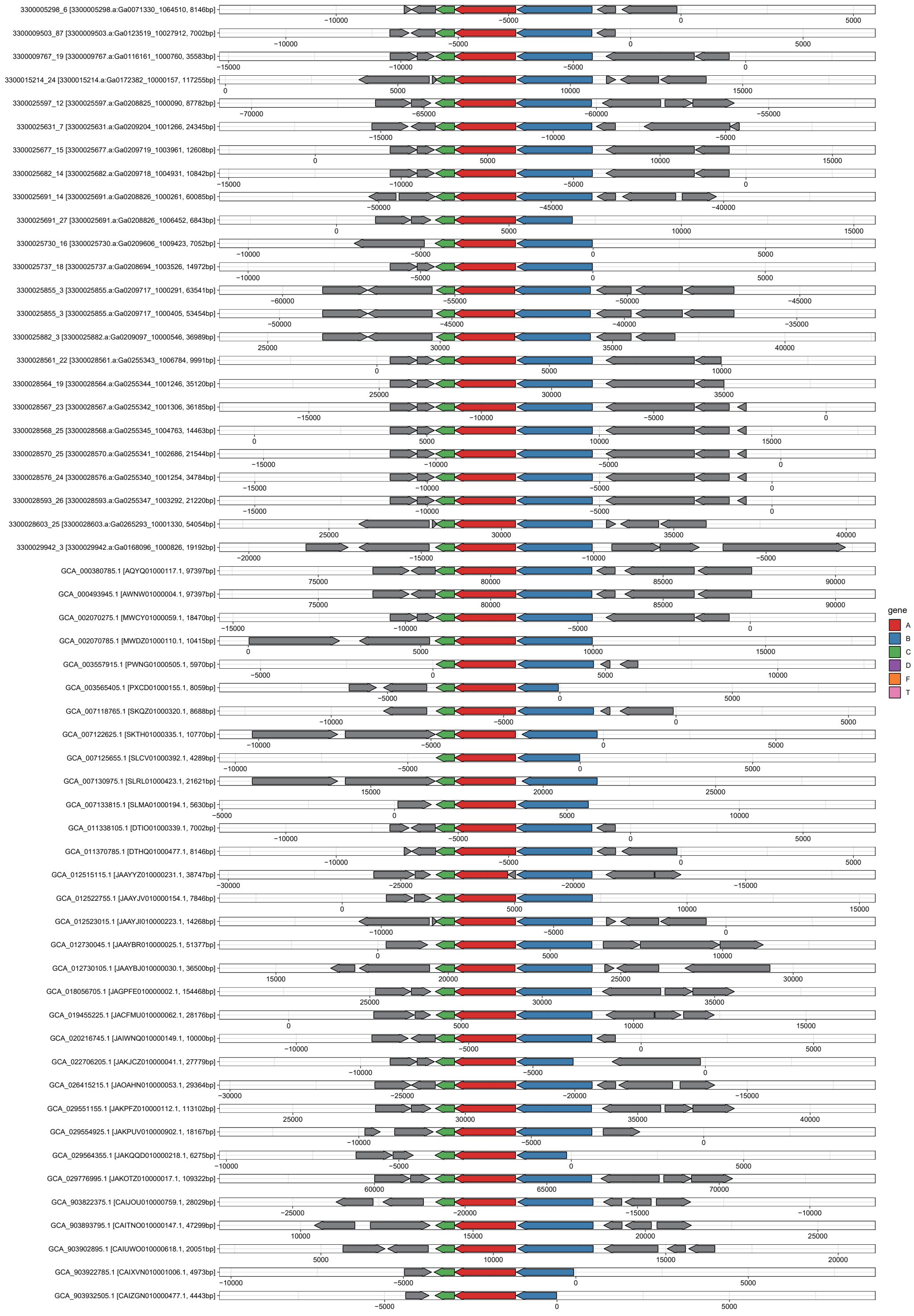
3300005298_6 [3300005298.a:Ga0071330_1064510, 8146bp]
3300009503_87 [3300009503.a:Ga0123519_10027912, 7002bp]
3300009767_19 [3300009767.a:Ga0116161_1000760, 35583bp]
3300015214_24 [3300015214.a:Ga0172382_10000157, 117255bp]
3300025597_12 [3300025597.a:Ga0208825_1000090, 87782bp]
3300025631_7 [3300025631.a:Ga0209204_1001266, 24345bp]
3300025677_15 [3300025677.a:Ga0209719_1003961, 12608bp]
3300025682_14 [3300025682.a:Ga0209718_1004931, 10842bp]
3300025691_14 [3300025691.a:Ga0208826_1000261, 60085bp]
3300025691_27 [3300025691.a:Ga0208826_1006452, 6843bp]
3300025730_16 [3300025730.a:Ga0209606_1009423, 7052bp]
3300025737_18 [3300025737.a:Ga0208694_1003526, 14972bp]
3300025855_3 [3300025855.a:Ga0209717_1000291, 63541bp]
3300025855_3 [3300025855.a:Ga0209717_1000405, 53454bp]
3300025882_3 [3300025882.a:Ga0209097_10000546, 36989bp]
3300028561_22 [3300028561.a:Ga0255343_1006784, 9991bp]
3300028564_19 [3300028564.a:Ga0255344_1001246, 35120bp]
3300028567_23 [3300028567.a:Ga0255342_1001306, 36185bp]
3300028568_25 [3300028568.a:Ga0255345_1004763, 14463bp]
3300028570_25 [3300028570.a:Ga0255341_1002686, 21544bp]
3300028576_24 [3300028576.a:Ga0255340_1001254, 34784bp]
3300028593_26 [3300028593.a:Ga0255347_1003292, 21220bp]
3300028603_25 [3300028603.a:Ga0265293_10001330, 54054bp]
3300029942_3 [3300029942.a:Ga0168096_1000826, 19192bp]
GCA_000380785.1 [AQYQ01000117.1, 97397bp]
GCA_000493945.1 [AWNW01000004.1, 97397bp]
GCA_002070275.1 [MWCY01000059.1, 18470bp]
GCA_002070785.1 [MWDZ01000110.1, 10415bp]
GCA_003557915.1 [PWNG01000505.1, 5970bp]
GCA_003565405.1 [PXCD01000155.1, 8059bp]
GCA_007118765.1 [SKQZ01000320.1, 8688bp]
GCA_007122625.1 [SKTH01000335.1, 10770bp]
GCA_007125655.1 [SLCV01000392.1, 4289bp]
GCA_007130975.1 [SLRL01000423.1, 21621bp]
GCA_007133815.1 [SLMA01000194.1, 5630bp]
GCA_011338105.1 [DTIO01000339.1, 7002bp]
GCA_011370785.1 [DTHQ01000477.1, 8146bp]
GCA_012515115.1 [JAAYYZ010000231.1, 38747bp]
GCA_012522755.1 [JAAYJV010000154.1, 7846bp]
GCA_012523015.1 [JAAYJI010000223.1, 14268bp]
GCA_012730045.1 [JAAYBR010000025.1, 51377bp]
GCA_012730105.1 [JAAYBJ010000030.1, 36500bp]
GCA_018056705.1 [JAGPFE010000002.1, 154468bp]
GCA_019455225.1 [JACFMU010000062.1, 28176bp]
GCA_020216745.1 [JAIWNQ010000149.1, 10000bp]
GCA_022706205.1 [JAKJCZ010000041.1, 27779bp]
GCA_026415215.1 [JAOAHN010000053.1, 29364bp]
GCA_029551155.1 [JAKPFZ010000112.1, 113102bp]
GCA_029554925.1 [JAKPUV010000902.1, 18167bp]
GCA_029564355.1 [JAKQQD010000218.1, 6275bp]
GCA_029776995.1 [JAKOTZ010000017.1, 109322bp]
GCA_903822375.1 [CAIJOU010000759.1, 28029bp]
GCA_903893795.1 [CAITNO010000147.1, 47299bp]
GCA_903902895.1 [CAIUWO010000618.1, 20051bp]
GCA_903922785.1 [CAIXVN010001006.1, 4973bp]
GCA_903932505.1 [CAIZGN010000477.1, 4443bp]
gene
A
B
C
D
F
T

**d**

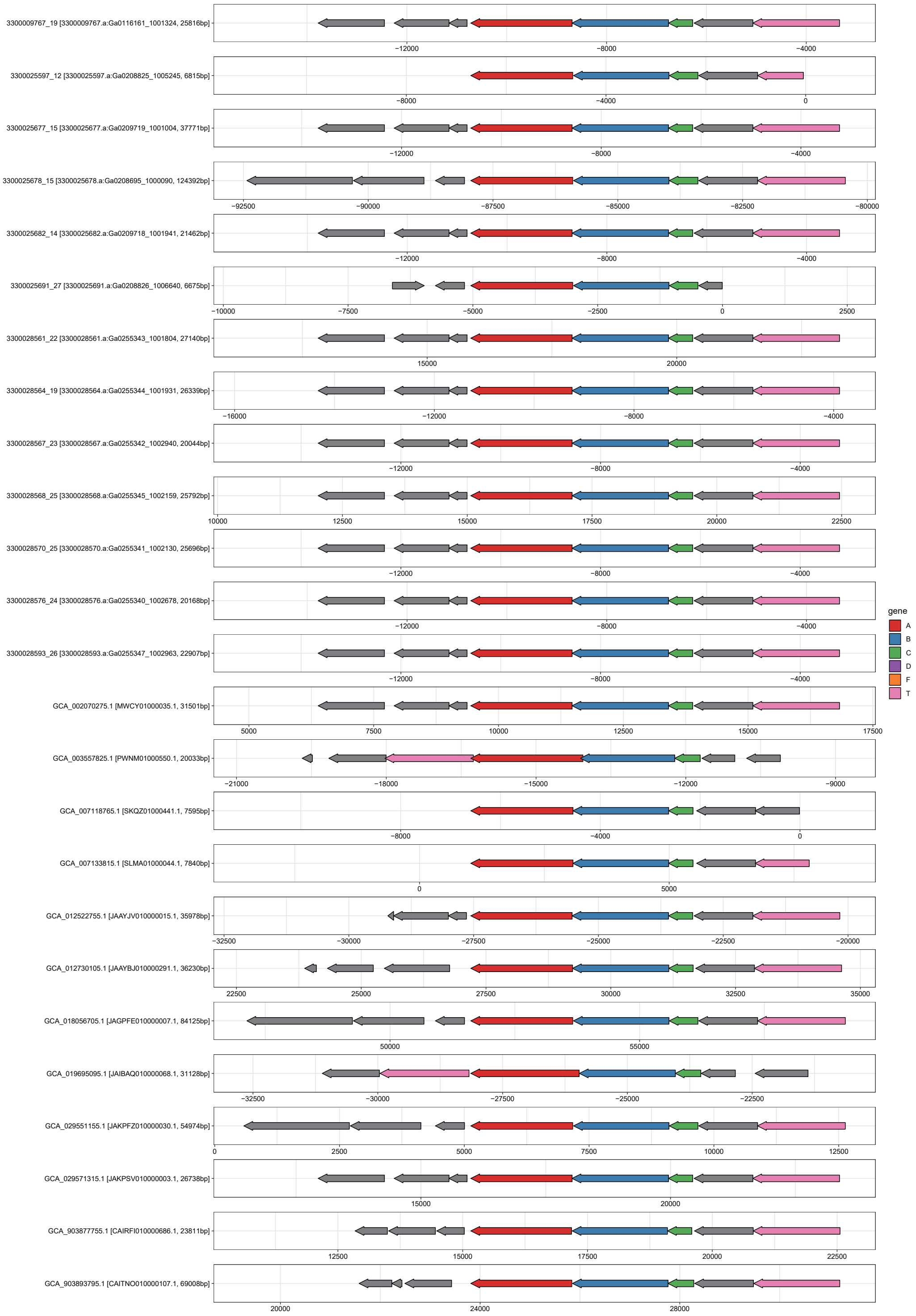
3300009767_19 [3300009767.a:Ga0116161_1001324, 25816bp]
−12000 −8000 −4000
3300025597_12 [3300025597.a:Ga0208825_1005245, 6815bp]
−8000 −4000 0
3300025677_15 [3300025677.a:Ga0209719_1001004, 37771bp]
−12000 −8000 −4000
3300025678_15 [3300025678.a:Ga0208695_1000090, 124392bp]
−92500 −90000 −87500 −85000 −82500 −80000
3300025682_14 [3300025682.a:Ga0209718_1001941, 21462bp]
−12000 −8000 −4000
3300025691_27 [3300025691.a:Ga0208826_1006640, 6675bp]
−10000 −7500 −5000 −2500 0 2500
3300028561_22 [3300028561.a:Ga0255343_1001804, 27140bp]
15000 20000
3300028564_19 [3300028564.a:Ga0255344_1001931, 26339bp]
−16000 −12000 −8000 −4000
3300028567_23 [3300028567.a:Ga0255342_1002940, 20044bp]
−12000 −8000 −4000
3300028568_25 [3300028568.a:Ga0255345_1002159, 25792bp]
10000 12500 15000 17500 20000 22500
3300028570_25 [3300028570.a:Ga0255341_1002130, 25696bp]
−12000 −8000 −4000
3300028576_24 [3300028576.a:Ga0255340_1002678, 20168bp]
−12000 −8000 −4000
3300028593_26 [3300028593.a:Ga0255347_1002963, 22907bp]
−12000 −8000 −4000
GCA_002070275.1 [MWCY01000035.1, 31501bp]
5000 7500 10000 12500 15000 17500
GCA_003557825.1 [PWNM01000550.1, 20033bp]
−21000 −18000 −15000 −12000 −9000
GCA_007118765.1 [SKQZ01000441.1, 7595bp]
−8000 −4000 0
GCA_007133815.1 [SLMA01000044.1, 7840bp]
0 5000
GCA_012522755.1 [JAAYJV010000015.1, 35978bp]
−32500 −30000 −27500 −25000 −22500 −20000
GCA_012730105.1 [JAAYBJ010000291.1, 36230bp]
22500 25000 27500 30000 32500 35000
GCA_018056705.1 [JAGPFE010000007.1, 84125bp]
50000 55000
GCA_019695095.1 [JAIBAQ010000068.1, 31128bp]
−32500 −30000 −27500 −25000 −22500
GCA_029551155.1 [JAKPFZ010000030.1, 54974bp]
0 2500 5000 7500 10000 12500
GCA_029571315.1 [JAKPSV010000003.1, 26738bp]
15000 20000
GCA_903877755.1 [CAIRFI010000686.1, 23811bp]
12500 15000 17500 20000 22500
GCA_903893795.1 [CAITNO010000107.1, 69008bp]
20000 24000 28000
gene
A
B
C
D
F
T

**e**

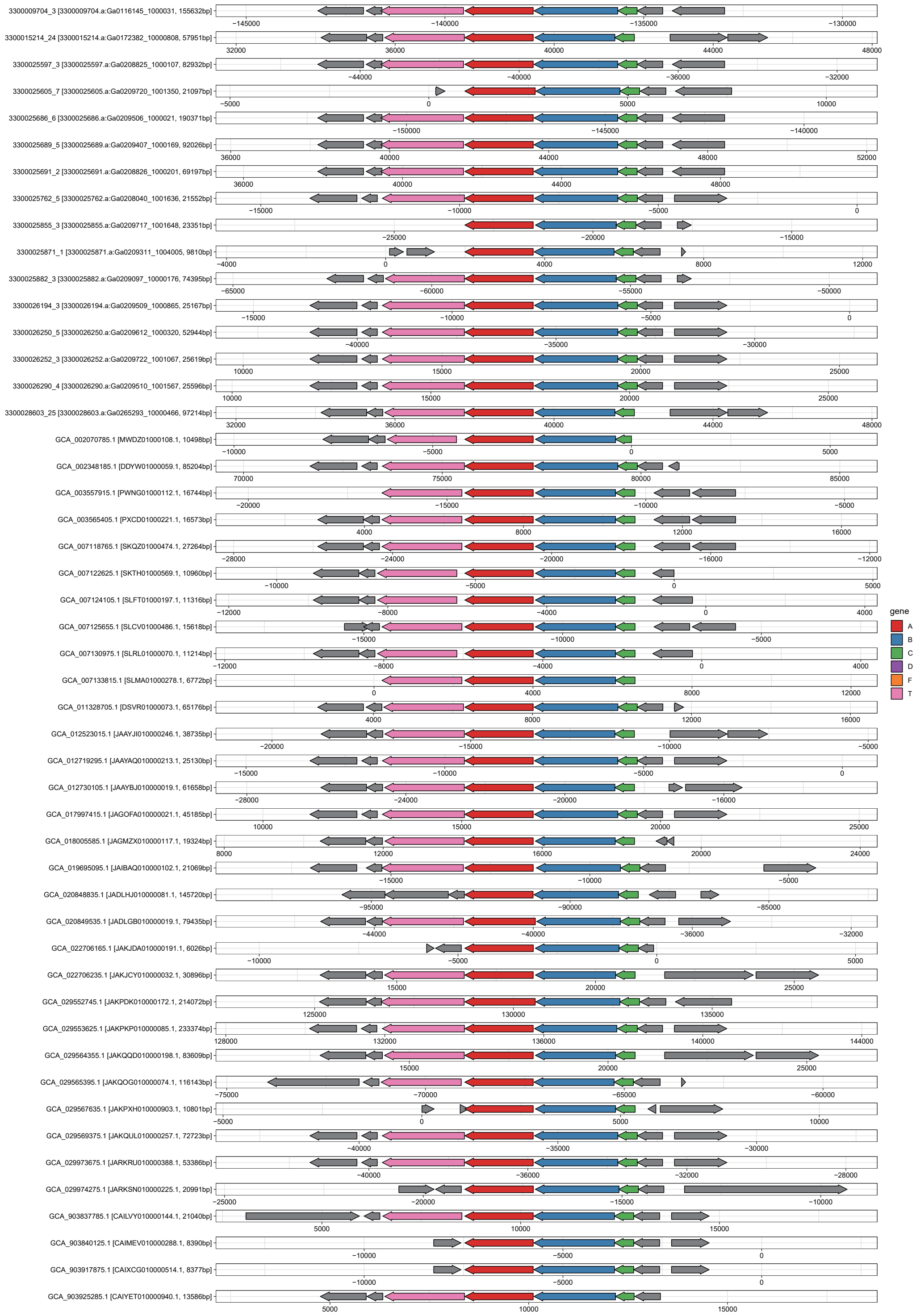

3300009704_3 [3300009704.a:Ga0116145_1000031, 155632bp]
3300015214_24 [3300015214.a:Ga0172382_10000808, 57951bp]
3300025597_3 [3300025597.a:Ga0208825_1000107, 82932bp]
3300025605_7 [3300025605.a:Ga0209720_1001350, 21097bp]
3300025686_6 [3300025686.a:Ga0209506_1000021, 190371bp]
3300025689_5 [3300025689.a:Ga0209407_1000169, 92026bp]
3300025691_2 [3300025691.a:Ga0208826_1000201, 69197bp]
3300025762_5 [3300025762.a:Ga0208040_1001636, 21552bp]
3300025855_3 [3300025855.a:Ga0209717_1001648, 23351bp]
3300025871_1 [3300025871.a:Ga0209311_1004005, 9810bp]
3300025882_3 [3300025882.a:Ga0209097_10000176, 74395bp]
3300026194_3 [3300026194.a:Ga0209509_1000865, 25167bp]
3300026250_5 [3300026250.a:Ga0209612_1000320, 52944bp]
3300026252_3 [3300026252.a:Ga0209722_1001067, 25619bp]
3300026290_4 [3300026290.a:Ga0209510_1001567, 25596bp]
3300028603_25 [3300028603.a:Ga0265293_10000466, 97214bp]
GCA_002070785.1 [MWDZ01000108.1, 10498bp]
GCA_002348185.1 [DDYW01000059.1, 85204bp]
GCA_003557915.1 [PWNG01000112.1, 16744bp]
GCA_003565405.1 [PXCD01000221.1, 16573bp]
GCA_007118765.1 [SKQZ01000474.1, 27264bp]
GCA_007122625.1 [SKTH01000569.1, 10960bp]
GCA_007124105.1 [SLFT01000197.1, 11316bp]
GCA_007125655.1 [SLCV01000486.1, 15618bp]
GCA_007130975.1 [SLRL01000070.1, 11214bp]
GCA_007133815.1 [SLMA01000278.1, 6772bp]
GCA_011328705.1 [DSVR01000073.1, 65176bp]
GCA_012523015.1 [JAAYJI010000246.1, 38735bp]
GCA_012719295.1 [JAAYAQ010000213.1, 25130bp]
GCA_012730105.1 [JAAYBJ010000019.1, 61658bp]
GCA_017997415.1 [JAGOFA010000021.1, 45185bp]
GCA_018005585.1 [JAGMZX010000117.1, 19324bp]
GCA_019695095.1 [JAIBAQ010000102.1, 21069bp]
GCA_020848835.1 [JADLHJ010000081.1, 145720bp]
GCA_020849535.1 [JADLGB010000019.1, 79435bp]
GCA_022706165.1 [JAKJDA010000191.1, 6026bp]
GCA_022706235.1 [JAKJCY010000032.1, 30896bp]
GCA_029552745.1 [JAKPDK010000172.1, 214072bp]
GCA_029553625.1 [JAKPKP010000085.1, 233374bp]
GCA_029564355.1 [JAKQQD010000198.1, 83609bp]
GCA_029565395.1 [JAKQOG010000074.1, 116143bp]
GCA_029567635.1 [JAKPXH010000903.1, 10801bp]
GCA_029569375.1 [JAKQUL010000257.1, 72723bp]
GCA_029973675.1 [JARKRU010000388.1, 53386bp]
GCA_029974275.1 [JARKSN010000225.1, 20991bp]
GCA_903837785.1 [CAILVY010000144.1, 21040bp]
GCA_903840125.1 [CAIMEV010000288.1, 8390bp]
GCA_903917875.1 [CAIXCG010000514.1, 8377bp]
GCA_903925285.1 [CAIYET010000940.1, 13586bp]
gene
A
B
C
D
F
T
