# Supplementary material for: Diverse non-canonical electron bifurcating [FeFe]-hydrogenases of separate evolutionary origins in Hydrogenedentota
Source: mSystems. 2024 Aug 27;9(9):e00999-24. doi: 10.1128/msystems.00999-24 (PMC11406978; doi:10.1128/msystems.00999-24)

Figure S6. Unrooted radiation tree of the subunits BfuA (left), BfuB (middle) and BfuC (right) from 195 nearly complete Group A3 [FeFe]-hydrogenases

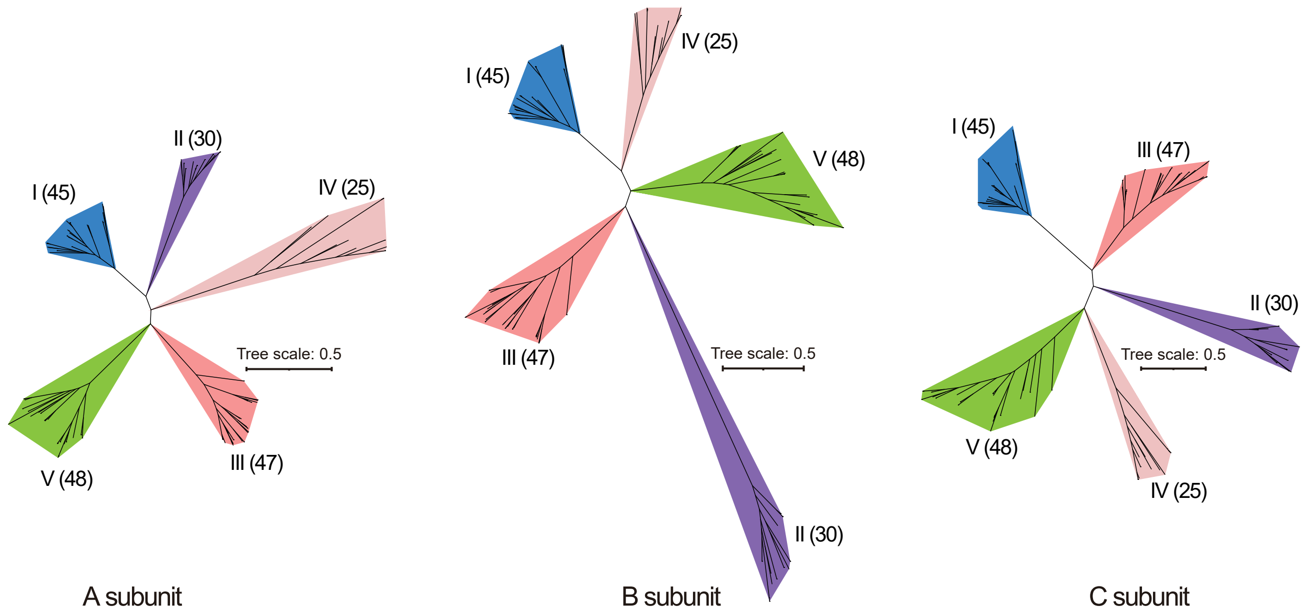

Supplement: Fig. S6 — Unrooted radiation tree of the subunits BfuA, BfuB, and BfuC. [file msystems.00999-24-s0008.pdf]
