# Supplementary material for: Diverse non-canonical electron bifurcating [FeFe]-hydrogenases of separate evolutionary origins in Hydrogenedentota
Source: mSystems. 2024 Aug 27;9(9):e00999-24. doi: 10.1128/msystems.00999-24 (PMC11406978; doi:10.1128/msystems.00999-24)

Figure S7. Schematic diagram indicating the extent of conservation in residues comprising the H-cluster motifs (P1, P2, and P3) of five sub-types of BfuA

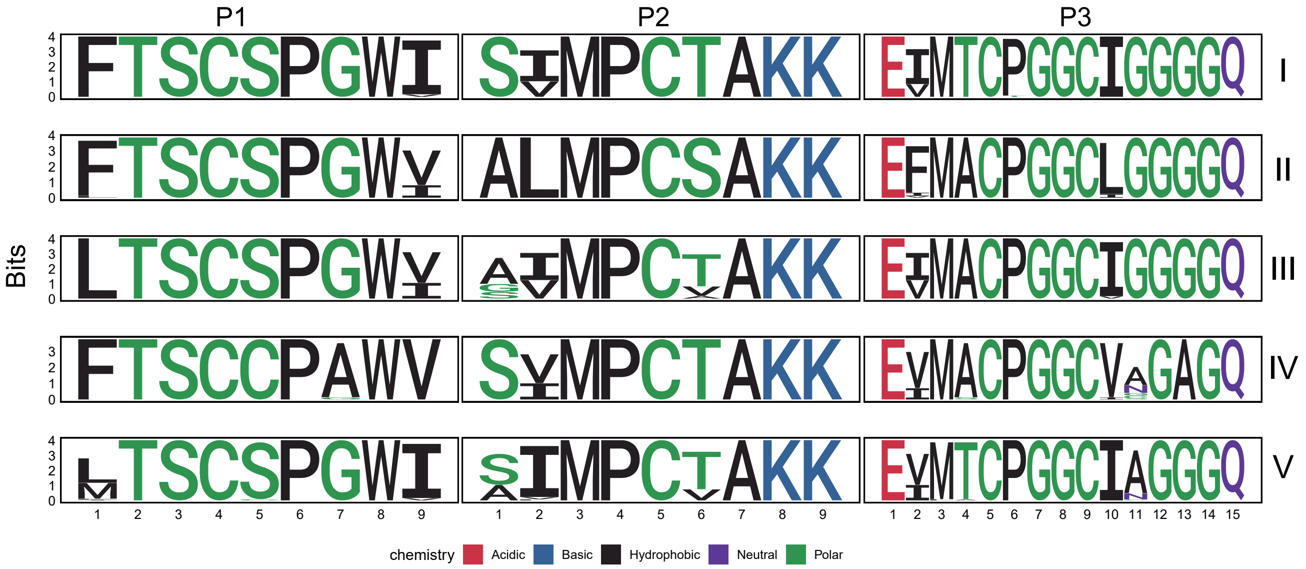

Supplement: Fig. S7 — Schematic diagram of H-cluster motifs. [file msystems.00999-24-s0009.pdf]
