# Supplementary material for: Diverse non-canonical electron bifurcating [FeFe]-hydrogenases of separate evolutionary origins in Hydrogenedentota
Source: mSystems. 2024 Aug 27;9(9):e00999-24. doi: 10.1128/msystems.00999-24 (PMC11406978; doi:10.1128/msystems.00999-24)

Figure S8. The boxplots represent events of inferred duplications, intra-phylum transfers, losses and gene originations during the evolution of *Hydrogenedentota*

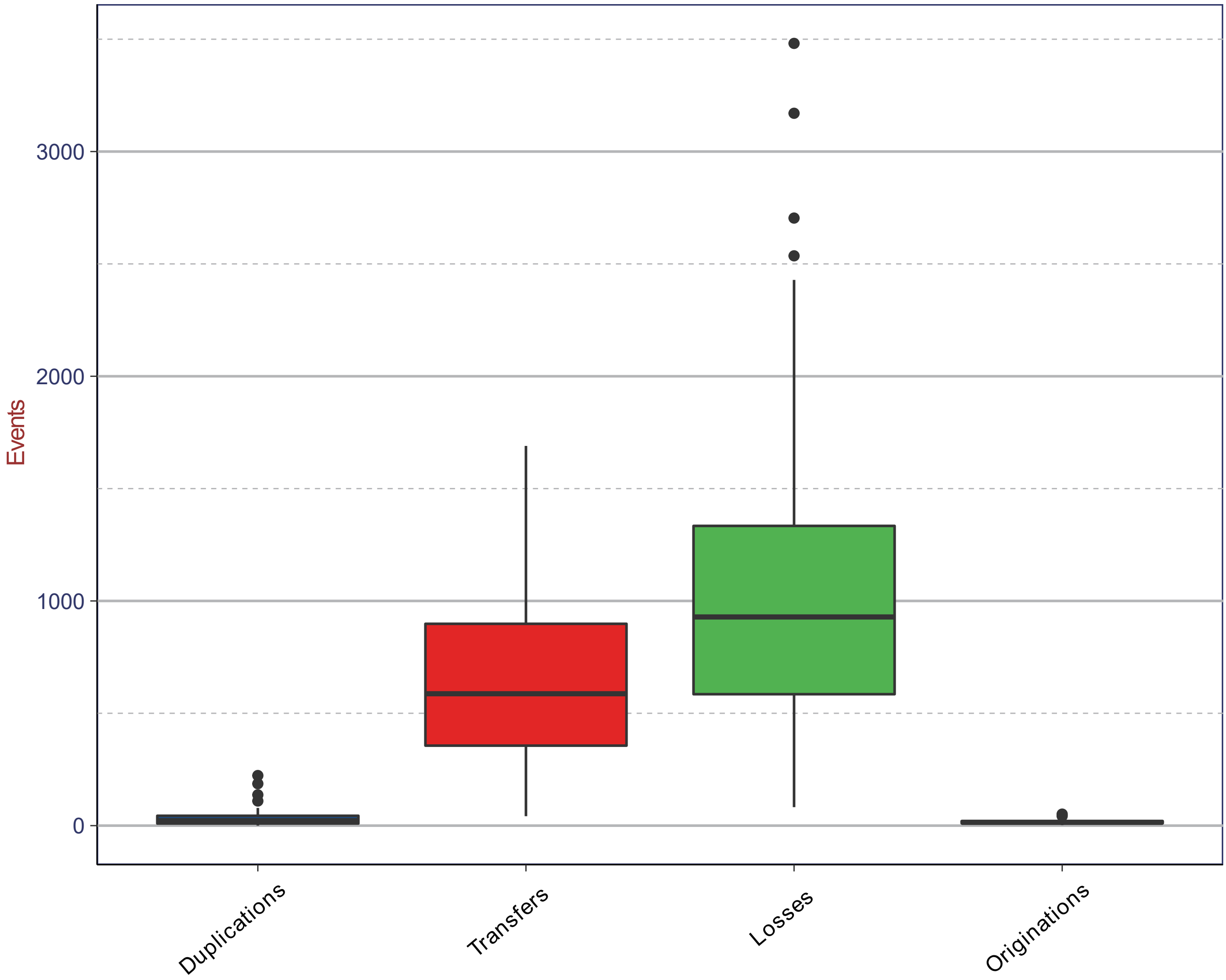

Supplement: Fig. S8 — Events of inferred duplications, intra-phylum transfers, losses and gene originations. [file msystems.00999-24-s0010.pdf]
